# Autohemotherapy combined with other external treatments of Traditional Chinese Medicine for chronic urticaria: a systematic review and meta-analysis

**DOI:** 10.3389/fmed.2026.1818960

**Published:** 2026-06-10

**Authors:** Jinchu Zhou, Boya Zhang, Haiying Liang, Xin Huang, Xingwei Liu, Yi Tong, Zhixuan Ai, Xinsheng Chen

**Affiliations:** 1 The Second Clinical College of Guangzhou University of Chinese Medicine, Guangzhou, China; 2 Guangdong Traditional Chinese Medicine Hospital, Guangzhou, China

**Keywords:** chronic urticaria, external therapy of TCM, meta-analysis, randomized controlled trial, systematic review

## Abstract

**Background:**

Chronic urticaria (CU) is a refractory, recurrent skin disease that adversely affects patients’ quality of life, mental health. Autohemotherapy (AHT) and various external treatments of Traditional Chinese Medicine (TCM) are reported to be effective in improving CU with few side effects. This study aims to evaluate the efficacy and safety of AHT combined with various TCM external treatments for CU.

**Methods:**

This study was conducted according to the Preferred Reporting Items for Systematic Reviews and Meta-Analyses (PRISMA) guidelines. 11 Chinese and English databases were searched from database inception to August 10, 2025, for randomized controlled trials (RCTs) on AHT combined with other TCM external treatments for CU. Two researchers independently completed literature screening and data extraction. Study quality was evaluated using the Cochrane Risk of Bias Tool 2.0 (RoB 2.0). Meta-analysis was performed using STATA 18.0.

**Results:**

A total of 20 studies involving 1,461 participants were included. The intervention group received AHT combined with other TCM external treatments, and the control group received Western medicine. The overall response rate was significantly better in the intervention group (RR: 1.17; 95% Confidence Interval, CI: 1.10–1.24; *p* < 0.001). Compared to the control group, the intervention group also showed significant improvements in recurrence rate (RR: 0.31; 95% CI: 0.23–0.42; *p* < 0.001), serum IgE level (SMD: −1.33; 95% CI: −1.76 to −0.91; *p* < 0.001), pruritus severity (SMD: −1.36; 95% CI: −2.17 to −0.54; *p* < 0.001), and wheal duration (SMD: −3.05; 95% CI: −6.05 to −0.06; *p* < 0.005). However, the results for pruritus severity and wheal duration were not robust and showed significant publication bias; therefore, their reliability is questionable.

**Conclusion:**

Autohemotherapy combined with some other TCM external treatments can improve clinical efficacy and reduce recurrence rates. However, due to concerns regarding unclear randomization and blinding, diverse outcome measures, methodological limitations, and high clinical heterogeneity among the included studies, more rigorous prospective studies are needed to conclusively evaluate its efficacy and safety.

**Systematic review registration:**

https://www.crd.york.ac.uk/PROSPERO/, CRD420261281760.

## 1 Introduction

Urticaria is a localized edema reaction caused by the widening of small blood vessels and increased permeability of the skin and mucosal blood vessels. It is marked by the appearance of wheals and associated pruritus. Chronic Urticaria (CU) is defined as wheals that last for more than 6 weeks, occurring intermittently or even daily. This condition seriously affects patients’ daily activities, work, quality of life, and mental health, imposing a substantial social burden (1–3). The incidence of CU in Asia is about 1.4%, which is higher than the rates reported in Europe (0.5%) and North America (0.1%) (4). The etiology of CU is complex. Modern medicine primarily uses second-generation H1-antihistamines as first-line treatment, which have a well-established safety profile with minimal adverse effects. However, more than one-third of patients fail to achieve adequate control with standard or increased doses (5), highlighting a clear unmet need for patients with recurrent attacks. Therefore, finding additional effective treatment options for patients who do not respond well to conventional therapy is both important and challenging.

Traditional Chinese Medicine (TCM) external treatments have a long history in managing CU. Modalities such as autohemotherapy (AHT), acupuncture, moxibustion, bloodletting, cupping therapy, acupoint thread-embedding, acupoint application, acupoint injection, auricular point therapy, and scraping therapy (Gua Sha) have demonstrated favorable therapeutic effects. In clinical practice, these external treatment methods are rarely used alone; they are most frequently applied in combination to enhance clinical efficacy.

AHT was developed by Professor Rui Jin (1932–2010) from Guangzhou University of Chinese Medicine. It involves extracting a small amount of the patient’s own venous blood and reinjecting it into specific acupoints or muscle tissue for the prevention and treatment of diseases. The autologous blood injection provides continuous, benign stimulation to the local area, which may produce an effect similar to specific immunotherapy (6), and its clinical efficacy is notable. A systematic review by Li Caicai and Wang Shaojun also supports the efficacy and safety of acupoint autologous blood acupoint injection for CU (7). In clinical practice, AHT is often combined with other TCM external treatments to manage CU. Numerous studies suggest that this combined approach may offer more advantages in promoting recovery, reducing adverse reactions, and lowering recurrence rates compared to traditional Western medicine alone. However, a comprehensive evidence-based medical evaluation is lacking. To more thoroughly assess the efficacy and safety of this combined therapy, this study conducted a meta-analysis of relevant domestic and international literature, aiming to provide an evidence-based reference for clinical practice.

## 2 Materials and methods

### 2.1 Study protocol

The study protocol was registered in the International Prospective Register of Systematic Reviews (PROSPERO, registration number: CRD420261281760). This study was conducted in accordance with the PRISMA (Preferred Reporting Items for Systematic Reviews and Meta-Analyses) 2020 guidelines (8).

### 2.2 Search strategy

Literature searches were performed using a combination of database-specific subject headings (e.g., MeSH) and free-text keywords. The following electronic databases were searched from their inception to August 10, 2,025: PubMed, Embase, The Cochrane Library, Web of Science, EBSCO, Chinese Biomedical Database (CBM), China National Knowledge Infrastructure (CNKI), Wan Fang Database, Chinese Scientific Journals Database (VIP), ClinicalTrials.gov, and the WHO International Clinical Trials Registry Platform (ICTRP). No language restrictions were applied. Chinese search terms included variations of: “自血疗法”“自体血液输注疗法”“自体血液注射疗法”“自血疗法肌内注射”“穴位自血注射”“自血穴位注射”“自体血穴位注射疗法”“经络注血疗法”“针刺”“揿针”“粗针”“腹针”“梅花针”“挑刺”“灸”“穴位埋线”“穴位贴敷”“穴位注射”“拔罐”“走罐”“线罐”“放血”“刮痧”“耳穴” and “慢性荨麻疹.” As the initial search employing “Chronic Urticaria” (and its free-text terms), “autohemotherapy,” and “autologous blood injection” in English databases did not warrant expansion, and to ensure a comprehensive search (i.e., to maximize recall), a comprehensive secondary search involving all TCM external therapy terms was not conducted. The detailed search strategy for PubMed is presented in Table 1. Authors of potentially relevant studies were contacted for missing information when necessary. Additionally, the reference lists of included articles were manually screened, and unpublished dissertations were sought. All retrieved records were imported into NoteExpress software to create a primary database, and duplicates were removed.

**Table 1:** Details of search strategy in PubMed.

Steps	Query
#1	(chronic urticaria[Mesh]) OR (chronic urticarias[Title/Abstract]) OR (urticaria, chronic[Title/Abstract]) OR (chronic spontaneous urticaria[Title/Abstract]) OR (chronic spontaneous urticarias[Title/Abstract]) OR (spontaneous urticaria, chronic[Title/Abstract]) OR (urticaria, chronic Spontaneous[Title/Abstract]) OR (idiopathic chronic urticaria[Title/Abstract]) OR (chronic urticaria, idiopathic[Title/Abstract]) OR (idiopathic chronic urticarias[Title/Abstract]) OR (urticaria, idiopathic chronic[Title/Abstract]) OR (chronic idiopathic urticaria[Title/Abstract]) OR (chronic idiopathic urticarias[Title/Abstract]) OR (idiopathic urticaria, chronic[Title/Abstract]) OR (urticaria, chronic idiopathic[Title/Abstract]) OR (chronic autoimmune urticaria[Title/Abstract]) OR (autoimmune urticaria, chronic[Title/Abstract]) OR (chronic autoimmune urticarias[Title/Abstract]) OR (urticaria, chronic autoimmune[Title/Abstract]) OR (autoimmune urticaria[Title/Abstract]) OR (autoimmune urticarias[Title/Abstract]) OR (urticaria, autoimmune[Title/Abstract])
#2	(autologous blood injection[Title/Abstract]) OR (autohemotherapy[Title/Abstract])
#3	#1 AND #2

### 2.3 Study selection

#### 2.3.1 Inclusion criteria

(1) Study Types: Randomized controlled trials (RCTs)
(2) Study Participants: Patients meeting the diagnostic criteria for CU (using specific or internationally recognized standards). Baseline characteristics (sex, age, disease duration) were required to be clearly reported and comparable between groups (*p* > 0.05). No restrictions were applied regarding age or sex.
(3) Intervention Measures: The intervention group received AHT combined with one or more other TCM external treatments. The control group received conventional Western medicine therapy.
(4) Outcome Indicators: Primary Outcome: Overall response rate, calculated as: (Total number of cases – Number of ineffective cases)/Total number of cases × 100%. Studies were required to clearly report the therapeutic outcomes (e.g., cured, markedly effective, effective, ineffective) along with the specific evaluation criteria used.

Secondary Outcomes: Serum Immunoglobulin E (IgE) level, recurrence rate, severity of pruritus, and duration of wheals.

#### 2.3.2 Exclusion criteria

The following were excluded: studies on AHT variants (e.g., ozone autohemotherapy, light quantum autohemotherapy, ultraviolet-irradiated and oxygenated autohemotherapy); case reports, animal experiments, and pharmacological studies; duplicate publications (only the earliest published version was retained); articles with suspected plagiarism or significant errors; and studies with unpublished, unavailable, or incomplete original data.

### 2.4 Literature screening and data extraction

Two researchers independently screened the titles, abstracts, and full texts of retrieved articles against the inclusion and exclusion criteria. Data were subsequently extracted after careful reading of the full texts and recorded using a pre-designed Excel form. Any inconsistencies were resolved through discussion with a third researcher. Extracted information included: (1) General information (first author, publication year, Geographical region, sample size); (2) Baseline data (sex, age, disease duration); (3) Treatment details (interventions in the experimental and control groups, treatment duration, follow-up time); (4) Outcome indicators; and (5) The number of adverse reactions.

### 2.5 Quality assessment of included literature

Two reviewers independently evaluated the methodological quality of the included RCTs using the Cochrane Risk of Bias Tool version 2.0 (RoB 2.0). Any disagreements during the assessment were resolved by discussion with a third reviewer. The tool assessed the following domains: bias arising from the randomization process, bias due to deviations from intended interventions, bias due to missing outcome data, bias in measurement of the outcome, and bias in selection of the reported result. The overall risk of bias for each study was judged as “low,” “some concerns,” or “high.”

### 2.6 Statistical methods

Statistical analysis was performed using STATA 18.0 software. For effect size analysis, the Risk Ratio (RR) with 95% Confidence Interval (CI) was used for dichotomous data (count data). Effect sizes were expressed as the risk ratio (RR) with 95% confidence intervals (CI) for dichotomous data, whereas the standardized mean difference (SMD) with 95% CI was used for continuous data due to variations in measurement scales or units across studies. Heterogeneity among studies was assessed using the Cochran’s Q test (with a significance level of *p* < 0.10) and the I^2^ statistic. If significant heterogeneity was present (*p* < 0.10 or I^2^ > 50%), a random-effects model was used for analysis. If heterogeneity was not statistically significant (*p* ≥ 0.10 and I^2^ ≤ 50%), a fixed-effect model was applied. In cases where the *p*-value from the *Q*-test was inconsistent with the I^2^ value, the I^2^ value was prioritized for determining the presence of heterogeneity. Subgroup analysis was conducted based on the different TCM external treatments used in the intervention groups. Sensitivity analysis was performed using the leave-one-out method. Publication bias for each outcome was analyzed using funnel plots and statistically tested with Harbord’s test (for count data) or Egger’s test (for measurement data).

### 2.7 Assessment of the quality of evidence

The Grading of Recommendations, Assessment, Development, and Evaluation (GRADE) approach was used to assess the quality of evidence for both primary and secondary outcomes (9).

## 3 Results

### 3.1 Literature search results

**Figure 1:**
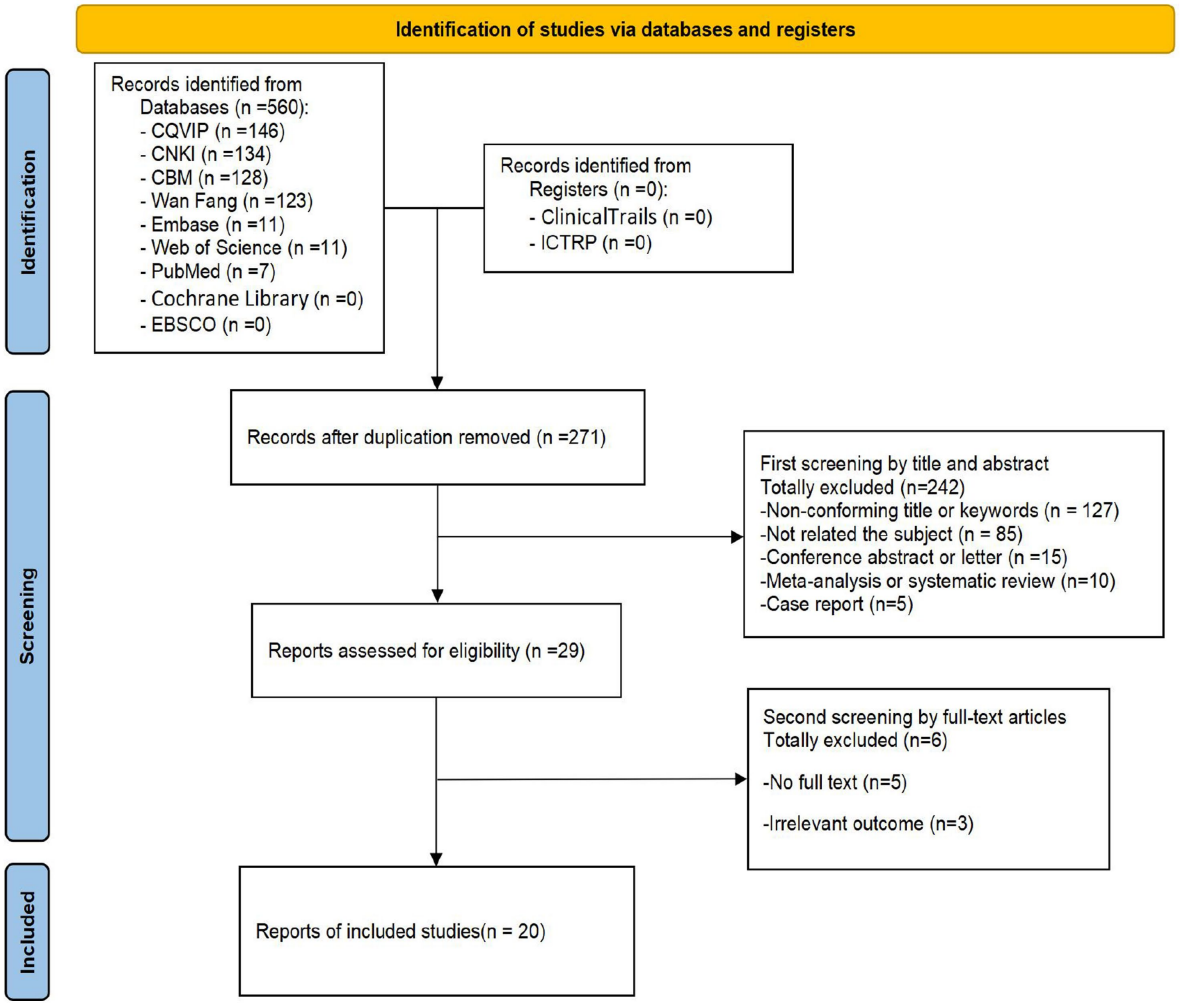
PRISMA flow diagram.

A total of 560 records were retrieved using the search strategy. After removing 289 duplicate records, 242 records were excluded based on title and abstract screening. The remaining 29 full-text articles were assessed for eligibility, resulting in 20 studies (10–29) being included in the final meta-analysis. The study selection process is illustrated in Figure 1.

### 3.2 Characteristics of included studies

A total of 1,461 participants were enrolled across the 20 included studies (9–28), with 696 in the intervention groups and 765 in the control groups. All intervention groups received AHT. The additional TCM therapies in the intervention groups were: acupuncture (5 studies) (14, 19, 20, 23, 29), moxibustion (3 studies) (12, 15, 17), acupoint catgut embedding therapy (3 studies) (21, 22, 26), acupoint-application (2 studies) (10, 11), apex auricular bloodletting (1 study) (18), and cupping therapy (5 studies) (13, 24, 25, 27, 28). One study (16) had two distinct intervention subgroups: one receiving acupoint catgut embedding combined with cupping, and the other receiving plum-blossom needle therapy combined with cupping (these were treated as separate groups in subsequent meta-analysis). All control groups were treated with conventional Western medicine. The characteristics of each included literature are shown in Table 2. Detailed descriptions of the specific operational procedures for each study are provided in Supplementary material 1.

**Table 2:** Basic characteristics of each included literature.

Study ID (Publication year)	Sample size (I/C, n)	Geographical region	Sex (male/female)	Age (year)	Disease course	Interventions		Treatment duration	Follow-up duration	Outcomes	Adverse events (I/C)
						I	C				
Peng et al. (14)	30/30	Nanjing, Jiangsu	NA	NA	NA	Acupuncture+Autohemotherapy	Loratadine 10 mg once daily	10wk	30d	①	0/0
Zhou et al. (29)	41/41	Foshan, Guangdong	I:24/17 C:23/18	I:(44.17 ± 3.15) C:(44.12 ± 2.14)	NA	Abdominal acupuncture+Autohemotherapy	Loratadine 10 mg once daily	30d	NA	①	NA
Qin (20)	33/33	Guangzhou, Guangong	I:15/18 C:14/19	I:(41.82 ± 12.02) C:(36.64 ± 10.78)	I:(30.27 ± 17.13)mo C:(30.73 ± 18.16)mo	Acupuncture+Autohemotherapy	Loratadine 10 mg once daily	30d	3mo	① ② ③ ④	1/30
Fang et al. (14)	21/20	Changsha, Hunan; Zhangjiajie, Hunan	I:13/8 C:9/11	I:(31 ± 8) C:(30 ± 8)	I:(7.03 ± 2.62)mo C:(7.46 ± 2.0)mo	Blood-nourishing and Wind-expelling NeedIing Method+Autohemotherapy	Cetirizine 10 mg once daily	1mo	2mo	①	0/0
Xu et al. (23)	30/30	Shenzhen, Guangdong	I:12/18 C:10/20	I:39.5 C:41	NA	Acupuncture+Autohemotherapy	Pheniramine 4 mg +Calcium Gluconate 2 g three times daily	4wk	NA	①	NA
Guo et al. (15)	40/40	Jinan, Shandong; Taian, Shandong	I:21/19 C:18/22	I:(32.4 ± 2.3) C:(31.8 ± 3.7)	I:(17.3 ± 0.6)wk. C:(17.6 ± 0.6)wk	Du moxibustion+Autohemotherapy	Epinastine hydrochloride 10 mg once daily	60d	6mo	① ③ ⑤	NA
Kang (17)	41/41	Panzhihua, Sichuan	I:23/18C:22/19	I:(35.31 ± 6.48) C:(34.45 ± 6.52)	NA	Medicated Blistering Moxibustion+Autohemotherapy	Mizolastine 10 mg once daily	40d	6mo	①	NA
Chen et al. (12)	40/40	Nanchang, Jiangxi	NA	NA	NA	Heat-sensitive Moxibustion+Autohemotherapy	Desloratadine citrate disodium 8.8 mg once daily	30d	1mo	① ② ⑤	NA
Su (21)	30/30	Nanning, Guangxi	I:12/18C:13/17	I:(31.07 ± 8.96)C:(27.67 ± 3.82)	I:(12.23 ± 6.80)mo C:(12.17 ± 7.27)mo	Catgut embedding therapy+Autohemotherapy	Loratadine 10 mg once daily	56d	3mo	① ②	1/1
Xie and Que (22)	68/68	Longyan, Fujian	I:21/47 C:23/45	I:(43. 8 ± 11.5) C:(44. 0 ± 12.1)	I:(5.18 ± 1.27)y C:(5.21 ± 1.30)y	Catgut embedding therapy+Autohemotherapy	Cetirizine 10 mg+ Ebastin 10 mg once daily	30d	NA	①	0/0
Zhang (26)	29/29	Xian, Shanxi	I:17/12 C:19/10	NA	NA	Catgut embedding therapy+Autohemotherapy	Desloratadine citrate disodium 8.8 mg once daily	50d	6mo	①	NA
Chen and Wu (11)	35/35	Ningde, Fujian	I:16/19C:17/18	I:(34.18 ± 10.43) C:(35.43 ± 9.82)	I:(16.18 ± 7.82)mo C:(16.83 ± 8.34)mo	Acupoint-application+Autohemo- therapy	Ebastin 10 mg once daily	6wk	3mo	① ②	NA
Chen (10)	30/30	Ningde, Fujian	I:14/16 C:13/17	I:(35.57 ± 11.49) C:(36.17 ± 9.89)	I:(16.40 ± 8.03)mo C:(17.13 ± 8.94)mo	Acupoint-application+Autohemo- therapy	Ebastin 10 mg once daily	6wk	1mo	① ② ③ ⑤	1/1
Liang et al. (18)	56/56	Gaozhou, Guangdong	I:29/27 C:25/31	I:(40. 81 ± 3.71) C:(40. 71 ± 3.91)	I:(3.55 ± 1.42)y C:(3.16 ± 1.81)y	Apex Auricular Bloodletting+Autohemotherapy	Loratadine 10 mg+ Ketotifen 10 mg once daily	4wk	NA	① ②	NA
Zhang et al. (24)	40/40	Chongqing	I:22/18 C:21/19	I:(50.50 ± 4.72) C:(52.00 ± 3.53)	NA	Moving cupping +Autohemotherapy	Cetirizine 10 mg once daily+ Dexamethasone acetate cream twice daily	30d	NA	① ③ ④ ⑤	NA
Zhao and Chen (28)	30/30	QIqihaer, Heilongjiang	I:14/16 C:15/15	I:(37.9 ± 12.44) C:(37.63 ± 13.12)	I:(2.09 ± 1.48)y C:(2.14 ± 1.69)y	Cupping therapy+Autohemotherapy	Levocetirizine 5 mg once daily	28d	NA	① ③	NA
Zhang and Lang (46)	52/52	Guangzhou, Guangdong; Beijing	I:24/28 C:23/29	I:(34 ± 11) C:(34 ± 11)	I:(1.3 ± 0.8)y C:(1.2 ± 0.9)y	Moving cupping +Autohemotherapy	Cetirizine 10 mg once daily+ Compound dexamethasone acetate cream once or twice daily	30d	3mo	① ⑤	NA
Ding (13)	30/31	Guangzhou, Guangdong	I:13/17C:11/20	I:(28.23 ± 6.78) C:(25.83 ± 5.21)	I:(21.97 ± 13.51)mo C:(23.00 ± 13.55)mo	Cupping therapy+ Autohemotherapy	Loratadine 10 mg once daily	4wk	2mo	① ② ③ ④	0/2
Zhang (27)	30/30	Haimen, Jiangsu	I:12/18 C:14/16	I:(30 ± 0.6) C:(31 ± 0.5)	I:(3.2 ± 0.8)mo C:(3.0 ± 0.9)mo	Acupoint Bloodletting Cupping+Autohemotherapy	Loratadine 10 mg once daily	60d	NA	①	NA
Jia et al. (16)	30/30	Lanzhou, Gansu	I_c_:13/17 I_p_:15/15 C:14/16	I_c_:(37.43 ± 5.27) I_p_:(38.1 ± 4.87) C:(36.53 ± 4.34)	I_c_:(21.07 ± 5.83)mo I_p_: (19.03 ± 4.39)mo C:(20.4 ± 5.43)mo	Catgut embedding therapy and Cupping/Plum-blossom Needle Cupping Therapy+Autohemotherapy	Ebastin 10 mg once daily	9wk	3mo	① ② ⑤	I_c_:0 I_p_:0 C:3

I, intervention group; C, control group; I_c_, The group treated with Catgut embedding therapy and Cupping + Autohemotherapy; I_p_, The group treated with Plum-blossom Needle Cupping Therapy+Autohemotherapy; d, day; wk, week; mo, month; y, year; ① Overall Response. Rate. ② Recurrence Rate. ③ Pruritus Severity. ④ Wheal Duration. ⑤ Serum IgE Level (IU/mL).

### 3.3 Risk of bias assessment

In the assessment of Domain 1 (randomization process), 11 studies (10, 11, 13–15, 19–21, 25, 28, 29) were rated as having a low risk of bias, where randomization sequence generation and allocation concealment were adequately described or were probably concealed. Four studies (16, 17, 22, 24) were rated as high risk due to inappropriate sequence generation methods (e.g., based on hospital number parity, order of registration, or order of visit). The remaining five studies (12, 18, 23, 26, 27) did not specify the method of sequence generation and concealment and were judged as having “some concerns.” Baseline characteristics were reported as comparable in all studies except for two (23, 24), which did not mention this information.

For Domain 2 (bias due to deviations from intended interventions), a high risk of bias was assigned to all studies, given the nature of the interventions and the lack of a suitable analytical method to adjust for deviations in adherence.

In Domains 3 (missing outcome data) and 5 (selection of the reported result), all 20 studies (10–29) were assessed as having a low risk of bias.

In Domain 4 (bias in measurement of the outcome), the outcome measures were considered to have been appropriately measured. However, none of the studies reported whether the outcome assessors were aware of the intervention received by participants. The outcomes in 13 studies (10, 12, 13, 16, 18, 20, 21, 25) were based on patient-rated scales or objective laboratory indicators, where the risk of assessment bias is considered lower. The outcomes in the remaining 7 studies (14, 22, 23, 26, 27) involved partly subjective assessments by clinicians, which introduces a potential risk of bias if assessors were aware of the treatment allocation.

**Figure 2:**
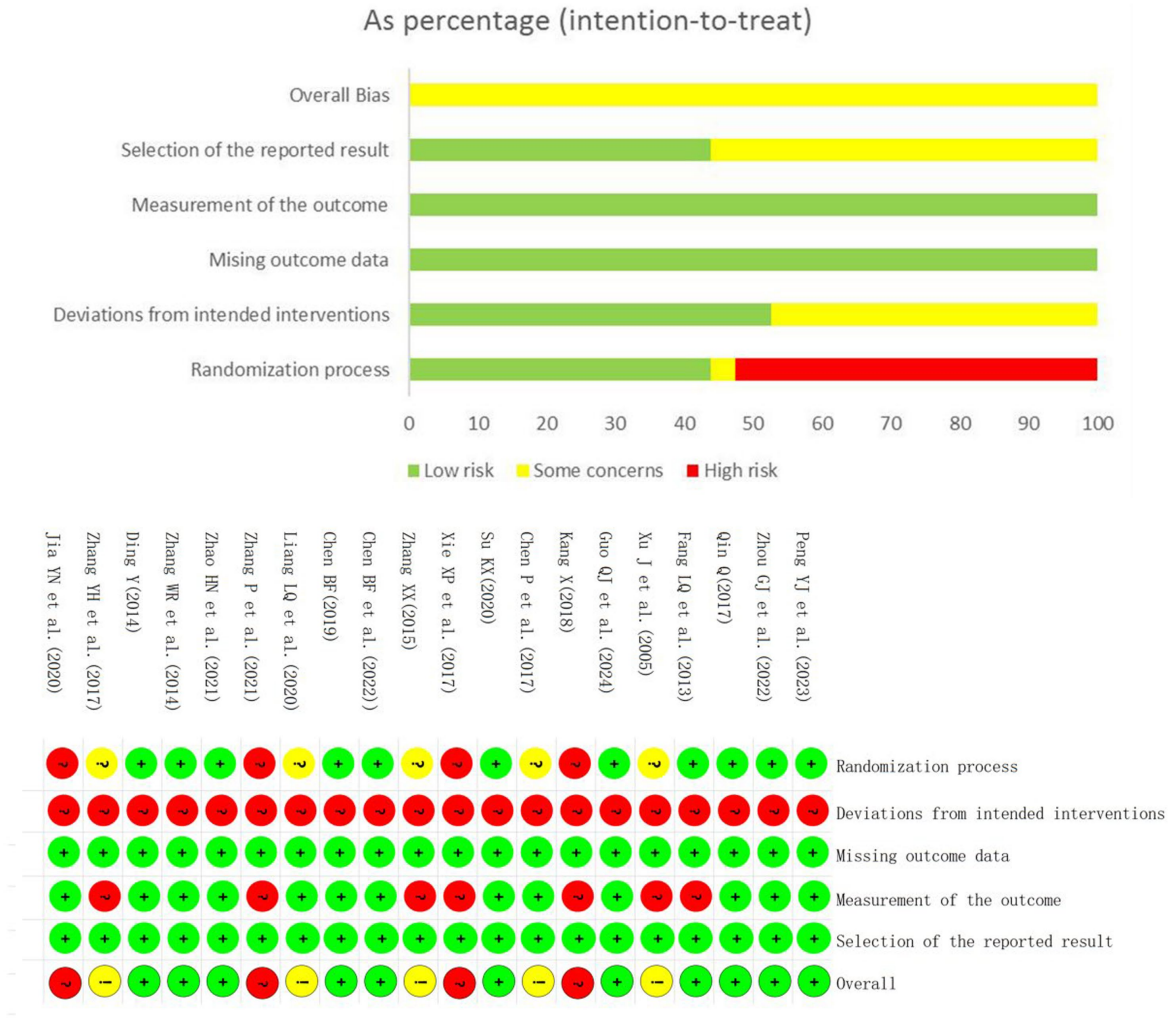
Risk of bias of each included study: red, high risk; green, low risk; yellow, unclear risk.

The results of the risk of bias analysis are summarized in Figure 2.

### 3.4 Results of meta-analysis

#### 3.4.1 Overall response rate

**Figure 3:**
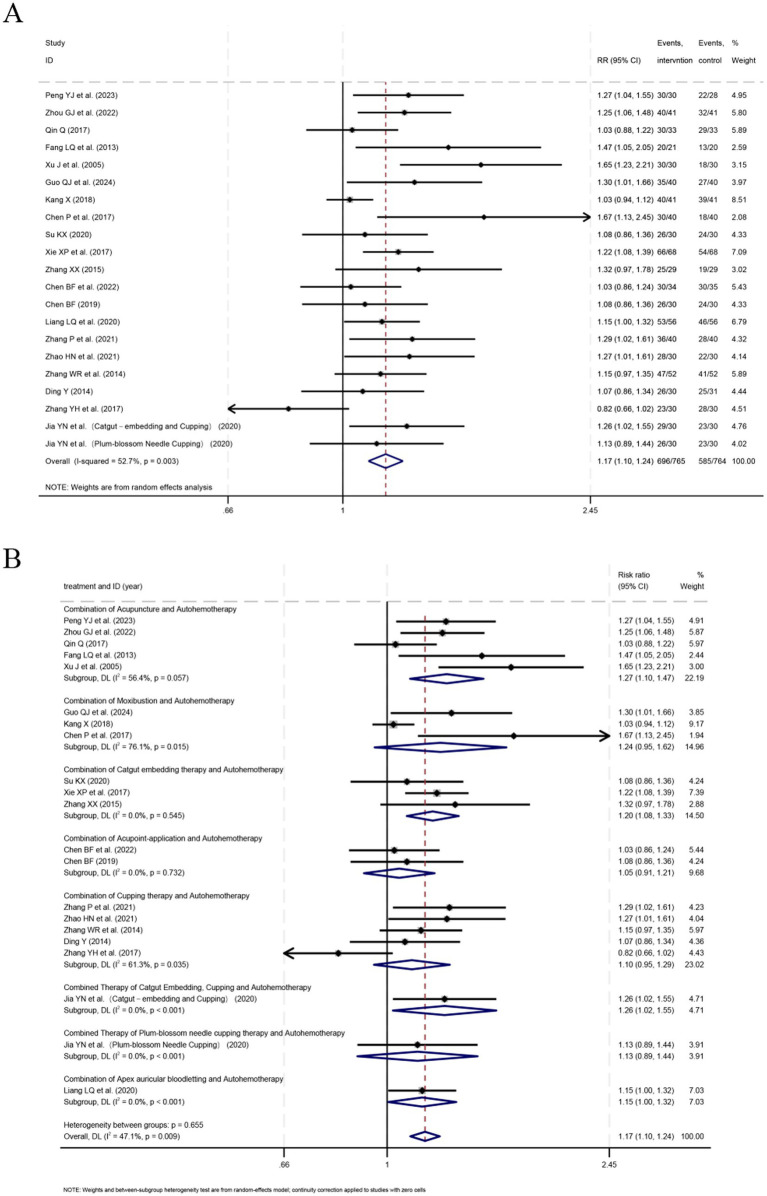
Forest plot of the overall response rate: pooled analysis **(A)** and subgroup analysis by specific therapeutic combinations **(B)**. CI, confidence interval.

**Figure 4:**
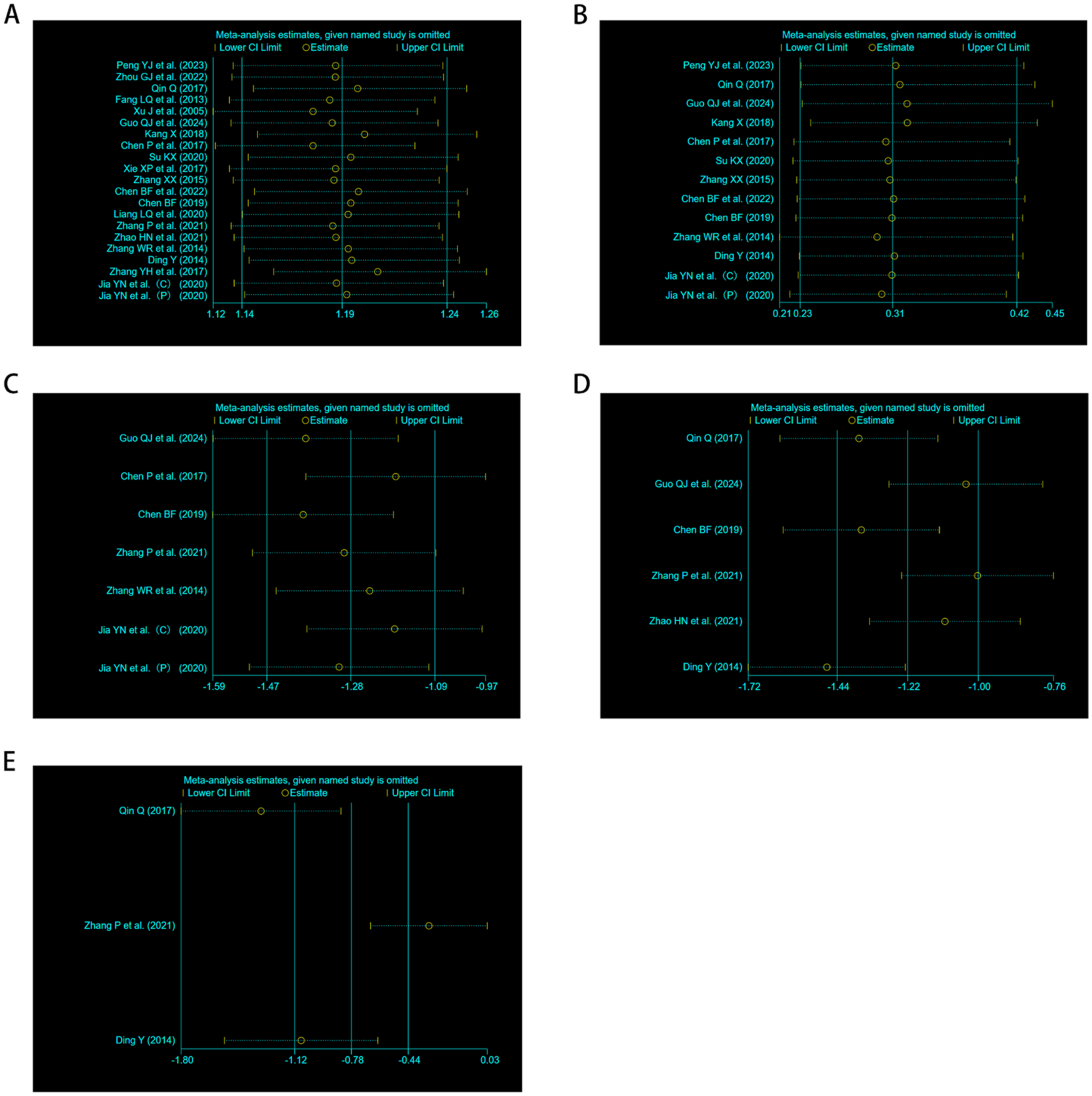
Sensitivity analysis results for five outcome indicators: overall response rate **(A)**, recurrence rate **(B)**, serum IgE level **(C)**, pruritus severity **(D)**, and wheal duration **(E)**.

**Figure 5:**
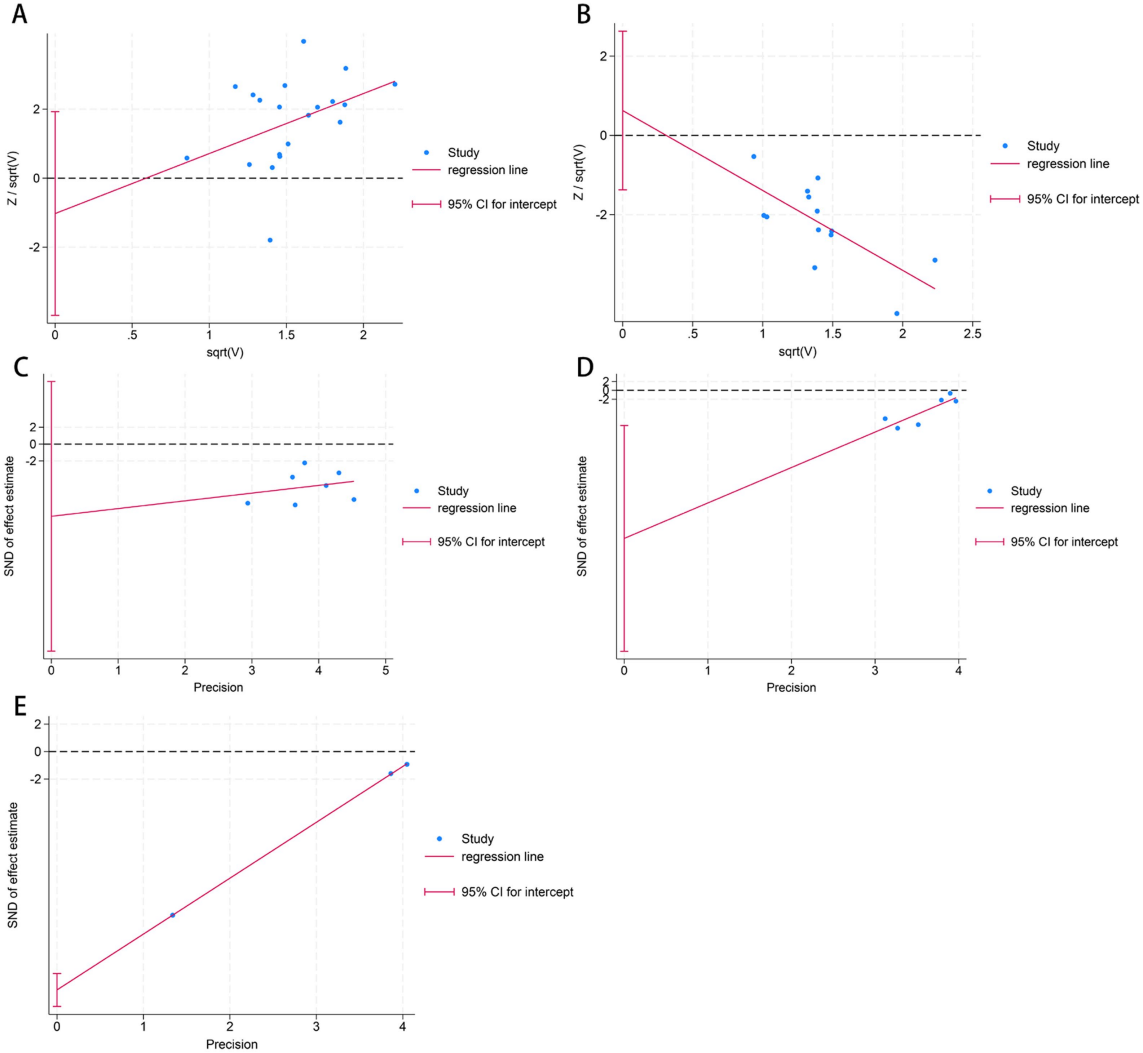
Publication bias diagrams for five outcome indicators: overall response rate **(A)**, recurrence rate **(B)**, serum IgE level **(C)**, pruritus severity **(D)**, and wheal duration **(E)**.

All 20 studies (9–28) compared the overall response rate between the treatment and control groups. Among them, 13 studies used urticaria symptom rating scales, though the specific scales varied. The scoring criteria of four studies (12, 16–18) were based on Mona Al-Ahmad (30) and EAACI/GA2LEN/EDF/WHO (2009, 2013) urticaria guidelines (31, 32), four studies (11, 15, 21, 29) used the “Guiding Principles for Clinical Research of New Chinese Medicines” (33), one (19) referred to “TCM Dermatology Syndrome Diagnostic and Efficacy Criteria” (34), and one (28) referred to “The Diagnostic and Efficacy Standard of TCM Syndromes” (35). The remaining 7 studies (14, 17, 22–24, 26, 27) evaluated efficacy subjectively and objectively based on changes in pruritus severity and wheal characteristics without using a formal scale. Due to the presence of heterogeneity among trials (*I*^2^ = 52.7%, *p* = 0.003), a random-effects model was used. The pooled analysis showed a statistically significant difference in the overall response rate favoring the intervention group (*p* < 0.001) (Figure 3A). Subgroup analysis based on the specific TCM therapy combined with AHT (Figure 3B) showed no significant difference in response rate for the moxibustion (*p* = 0.112), acupoint-application (*p* = 0.500), cupping therapy (*p* = 0.200), and plum-blossom needle cupping therapy (*p* = 0.321) subgroups. Significant differences were observed for the acupuncture (*p* = 0.001), catgut embedding therapy (*p* = 0.001), catgut embedding plus cupping (*p* = 0.029), and apex auricular bloodletting (*p* = 0.043) subgroups, although heterogeneity remained high in the acupuncture subgroup. Univariate meta-regression on treatment duration did not explain the heterogeneity (*p* = 0.918). Sensitivity analysis showed stable results (Figure 4A). Harbord’s test indicated no significant publication bias (*p* = 0.476) (Figure 5A). But the reliability of the overall response rate results for the acupuncture subgroup is limited as the source of heterogeneity could not be identified. The results of the other subgroups were relatively robust.

#### 3.4.2 Recurrence rate

**Figure 6:**
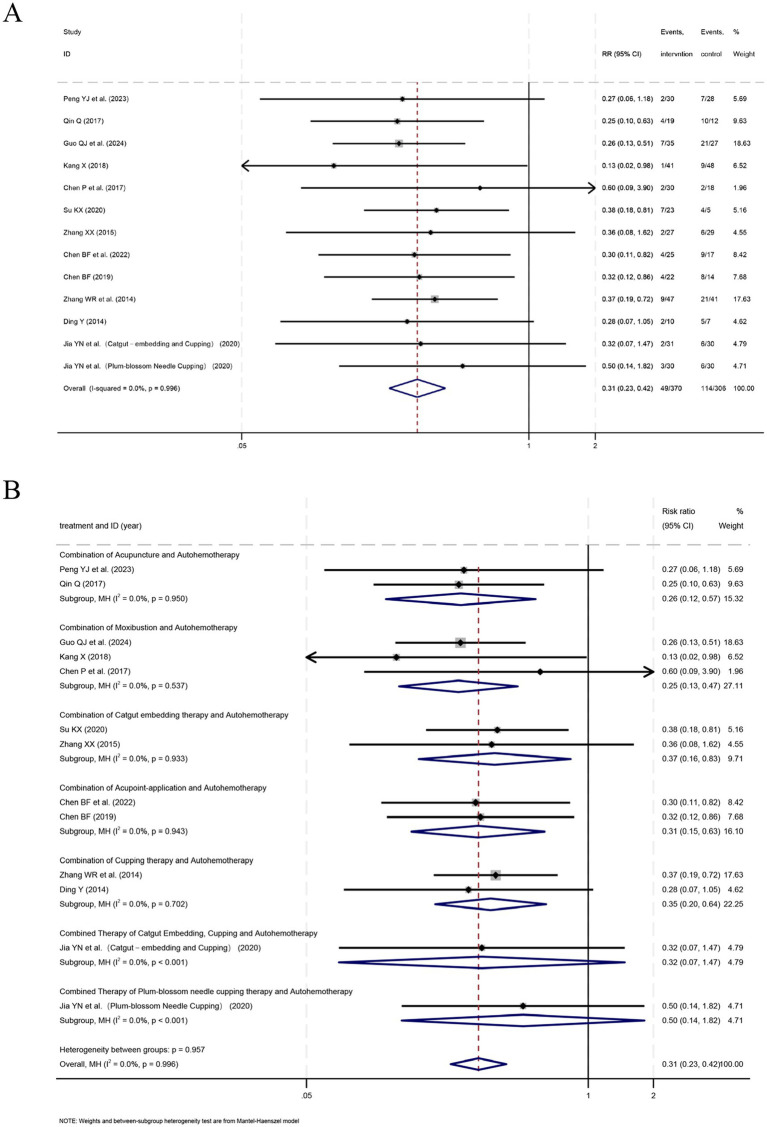
Forest plot of the recurrence rate: pooled analysis **(A)** and subgroup analysis by specific therapeutic combinations **(B)**. CI, confidence interval.

A total of 12 studies (10, 12, 13, 15–17, 19–21, 25, 26) reported recurrence rates. As heterogeneity among these studies was not significant (I^2^ = 0%, *p* = 0.996), a fixed-effect model was used. The results showed a statistically significant difference in the overall recurrence rate between groups, favoring the intervention (*p* < 0.001) (Figure 6A). Subgroup analysis based on the specific therapeutic combinations (Figure 6B) revealed that the response rate was significantly improved in the acupuncture (*p* = 0.001), moxibustion (*p* < 0.001), catgut embedding therapy (*p* = 0.016), acupoint-application (*p* = 0.001), and cupping therapy (*p* = 0.001) subgroups, whereas no significant improvement was found in the catgut embedding plus cupping (*p* = 0.144) or plum-blossom needle cupping therapy (*p* = 0.292) subgroups. Sensitivity analysis of the 12 included studies showed that no single study significantly altered the overall result (Figure 4B). Harbord’s test showed no significant publication bias (*p* = 0.504) (Figure 5B), indicating that the analysis of recurrence rate is highly reliable and stable.

#### 3.4.3 Serum IgE level

**Figure 7:**
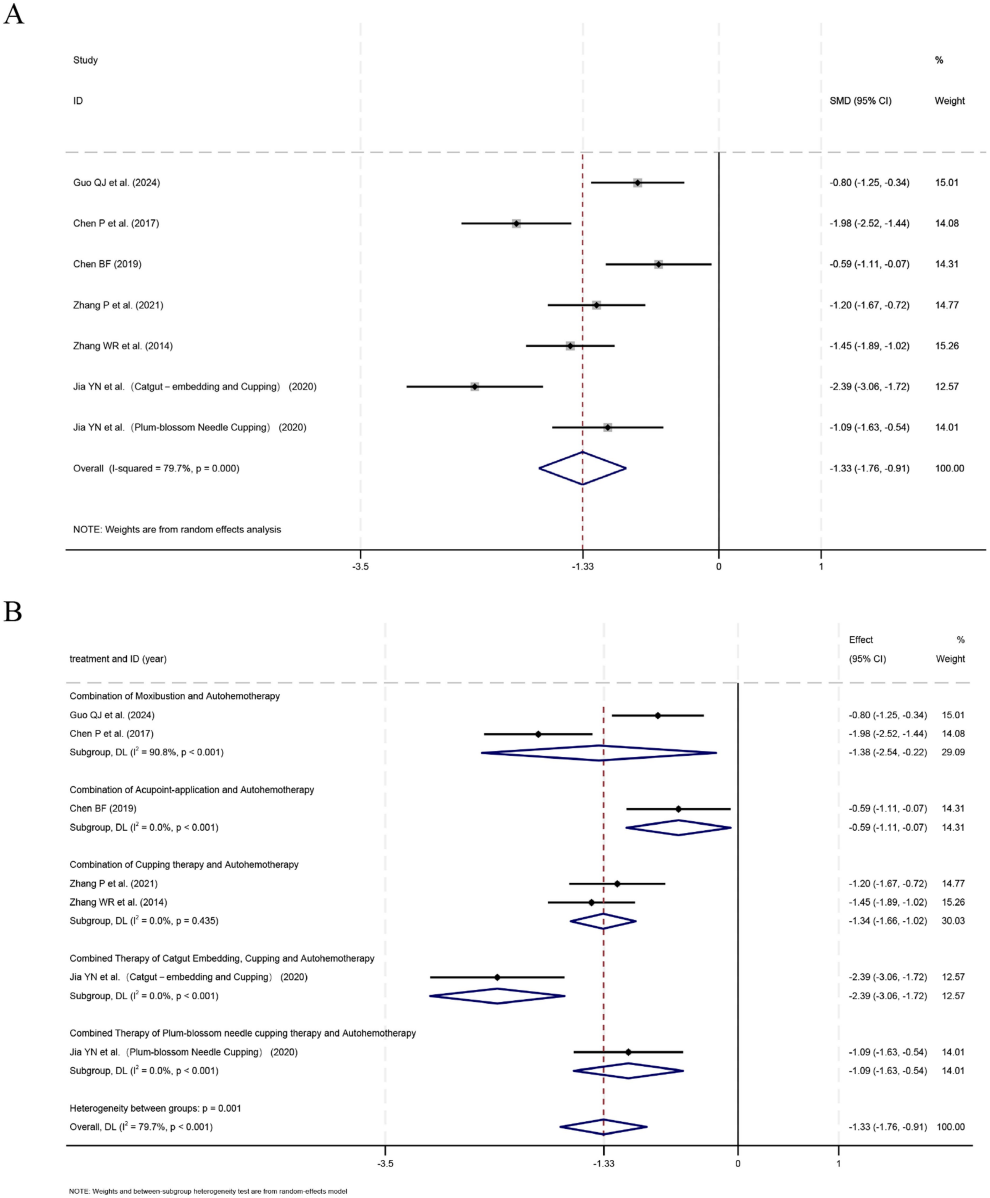
Forest plot of the serum IgE level: pooled analysis **(A)** and subgroup analysis by specific therapeutic combinations **(B)**. CI, confidence interval.

Six studies (10, 12, 15, 16, 24, 25) reported serum IgE levels. Due to significant heterogeneity (*I*^2^ = 79.7%, *p* < 0.001), a random-effects model was used. The pooled result showed a statistically significant difference between groups (*p* < 0.001) (Figure 7A). Subgroup analysis (Figure 7B) found no statistically significant difference for the acupoint-application subgroup (*p* = 0.025). Significant differences were observed for the moxibustion (*p* = 0.020), catgut embedding plus cupping (*p* < 0.001), and plum-blossom needle cupping therapy (*p* < 0.001) subgroups, though heterogeneity remained significant in the moxibustion subgroup. Subsequent sensitivity testing showed stable results (Figure 4C). Egger’s test indicated no significant publication bias (*p* = 0.227) (Figure 5C). Except for the moxibustion subgroup, the results of the other subgroups were relatively reliable. The observed heterogeneity may be attributed to differences in detection methods and kits across studies.

#### 3.4.4 Pruritus severity

**Figure 8:**
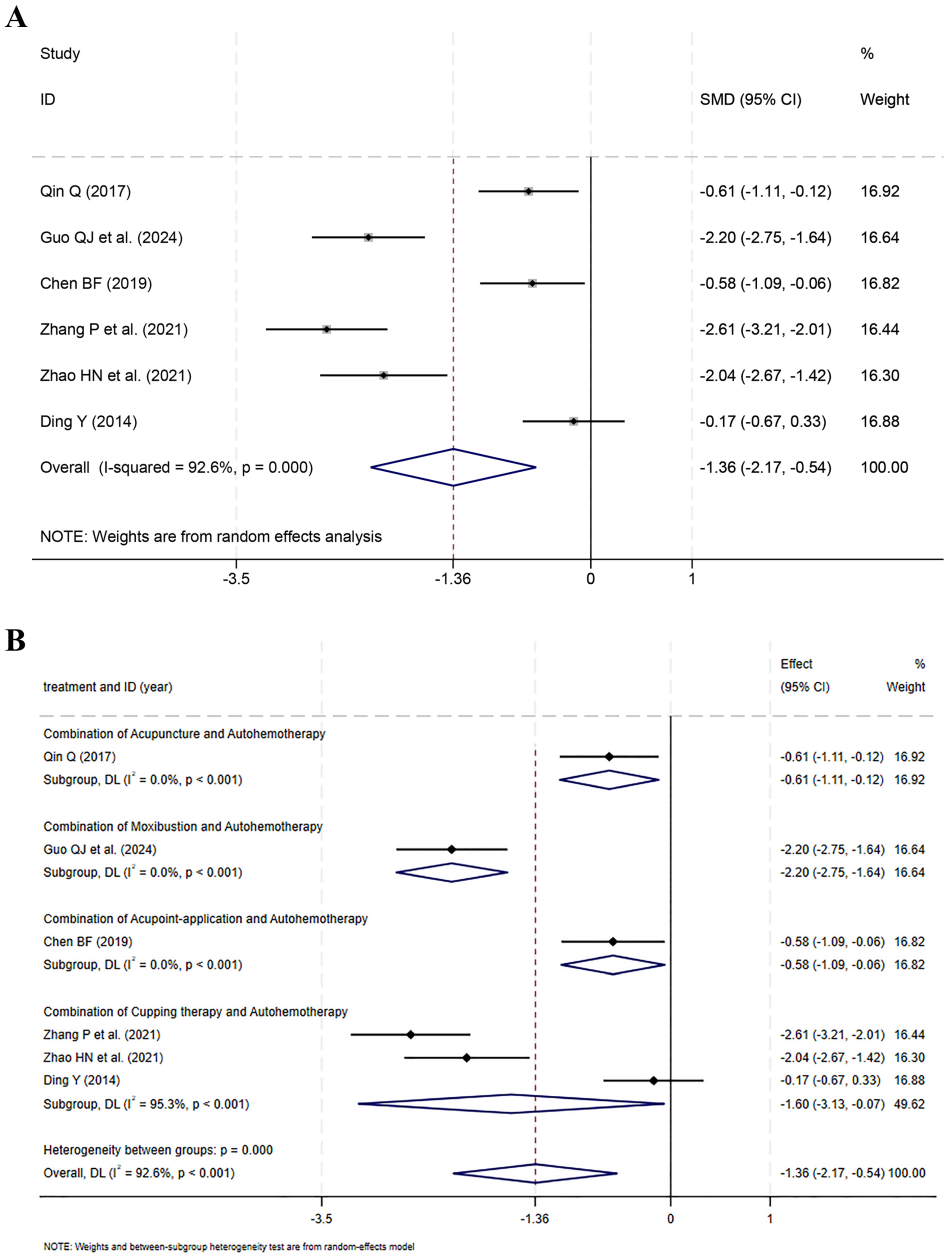
Forest plot of the pruritus severity: pooled analysis **(A)** and subgroup analysis by specific therapeutic combinations **(B)**. CI, confidence interval.

Six studies (10, 13, 15, 20, 24, 28) reported pruritus severity scores. Significant heterogeneity was present (*I*^2^ = 92.6%, *p* < 0.001), leading to the use of a random-effects model. The results indicated a statistically significant difference between groups (*p* < 0.001) (Figure 8A). Subgroup analysis (Figure 8B) showed significant differences for the acupuncture (*p* = 0.015), moxibustion (*p* < 0.001), acupoint-application (*p* = 0.029), and cupping therapy (*p* = 0.041) subgroups. Heterogeneity remained high in the cupping subgroup. Sensitivity analysis showed that the results became unstable after excluding one specific study (13) (Figure 4D). Even after removing this study, heterogeneity remained very high (*I*^2^ = 96.9%, *p* < 0.001). Egger’s test indicated significant publication bias (*p* = 0.022) (Figure 5D), thus the reliability of these results is questionable.

#### 3.4.5 Wheal duration

**Figure 9:**
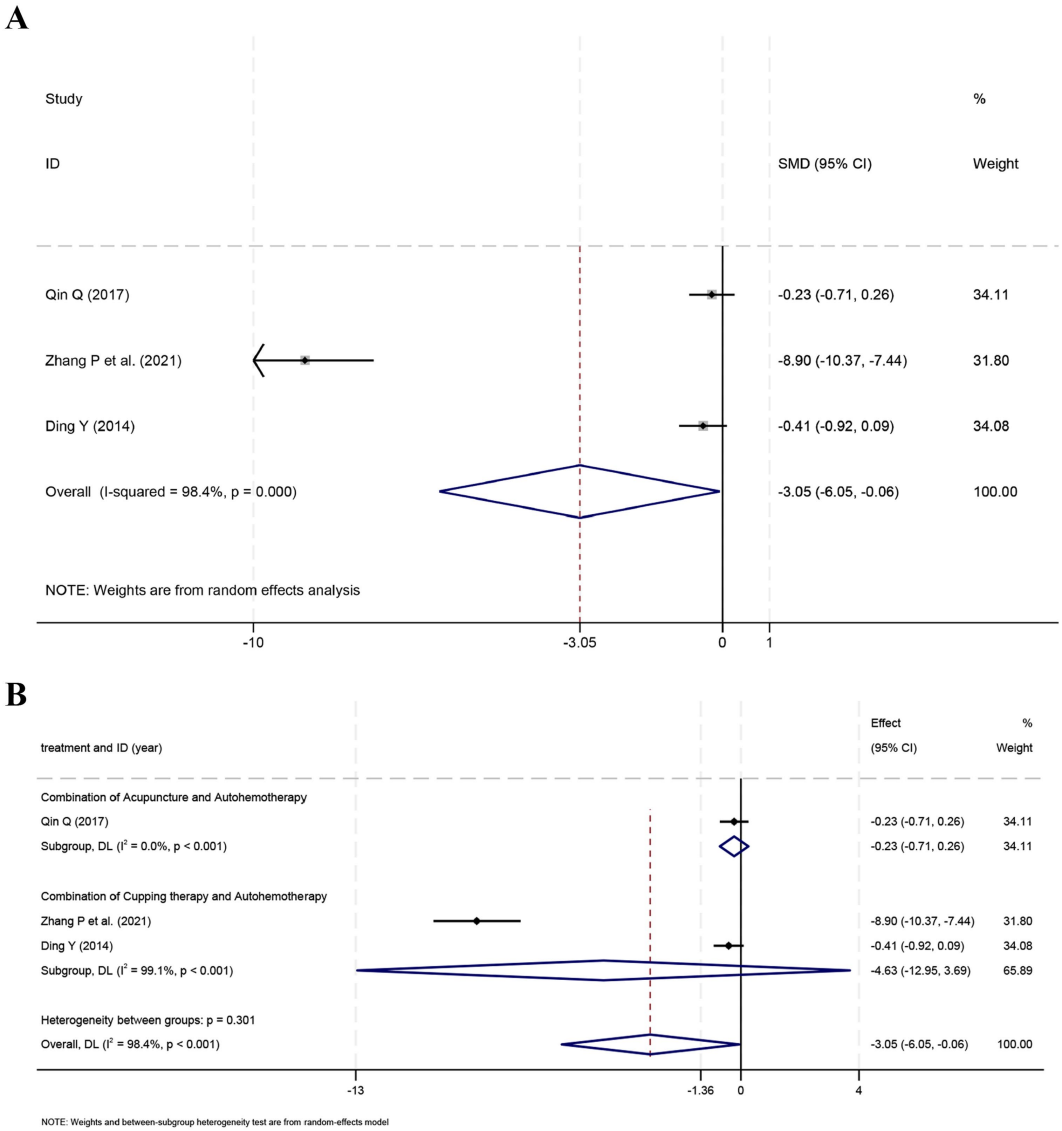
Forest plot of the wheal duration: pooled analysis **(A)** and subgroup analysis by specific therapeutic combinations **(B)**. CI, confidence interval.

Three studies (13, 20, 24) reported the duration of wheals. Due to extreme heterogeneity (*I*^2^ = 98.4%, *p* < 0.001), a random-effects model was applied. The results showed a statistically significant difference between groups (*p* < 0.005) (Figure 9A). Subgroup analysis (Figure 9B) found no significant difference for the acupuncture (*p* = 0.354) or cupping therapy (*p* = 0.275) subgroups. Heterogeneity remained significant in the cupping subgroup. Sensitivity analysis (Figure 4E) indicated that the results were unstable, influenced heavily by two studies (20, 24). Egger’s test showed significant publication bias (*p* = 0.003) (Figure 5E), further questioning the reliability of these findings.

#### 3.4.6 Adverse reactions

Five studies reported adverse effects. In one study (20), the control group experienced mild adverse reactions such as dry mouth, drowsiness, and fatigue, which resolved after treatment completion. One patient in the treatment group had slight dizziness that improved after a short rest. Another study (13) reported one case of mild dizziness and 1 case of dry mouth in the control group, which were self-limiting and did not interrupt treatment. A third study (16) noted two cases of mild sleepiness and 1 case of dizziness in the control group, which resolved naturally after drug cessation. Two other studies (10, 21) mentioned one adverse event occurring in each group but did not specify the symptoms.

#### 3.4.7 Certainty of evidence assessed using the GRADE approach

The GRADE certainty of evidence for the primary and secondary outcomes is presented in Table 3. The certainty of evidence was low for the recurrence rate and very low for the overall response rate, serum IgE levels, pruritus severity, and wheal duration.

**Table 3:** GRADE assessment of the included studies.

Certainty assessment							No. of patients		Effect		Certainty	Importance
No. of studies	Study design	Risk of bias	Inconsistency	Indirectness	Imprecision	Other considerations	Intervention group	Control group	Relative (95% CI)	Absolute (95% CI)		
Overall response rate												
20	Randomized trials	Serious^a^	Serious^b^	Not serious	Serious^c^	None	696/765 (91.0%)	585/764 (76.6%)	RR 1.17 (1.10 to 1.24)	130 more per 1,000 (from 77 more to 184 more)	⨁◯◯◯ Very low^a,b,c^	Critical
Recurrence rate												
12	Randomized trials	Serious^a^	Serious^b^	Not serious	Not serious	None	49/370 (13.2%)	114/306 (37.3%)	RR 0.31 (0.23 to 0.42)	257 fewer per 1,000 (from 287 fewer to 216 fewer)	⨁⨁◯◯ Low^a,b^	Important
Serum IgE level												
6	Randomized trials	Serious^a^	Serious^b^	Not serious	Serious^c^	None			SMD -1.33 (−1.76 to −0.91)	-- per 1,000 (from -- to --)	⨁◯◯◯ Very low^a,b,c^	Important
Pruritus severity												
6	Randomized trials	Serious^a^	Serious^b^	Not serious	Not serious	Publication bias strongly suspected			SMD -1.36 (−2.17 to −0.54)	-- per 1,000 (from -- to --)	⨁◯◯◯ Very low^a,b^	Important
Wheal duration												
3	Randomized trials	Serious^a^	Not serious	Not serious	Serious^c^	Publication bias strongly suspected			SMD -3.05 (−6.05 to −0.06)	-- per 1,000 (from -- to --)	⨁◯◯◯ Very low^a,c^	Important

CI, confidence interval; RR, risk ratio. ^a^Downgrade one level: All the research on intervention measures has specificities, and there are also unreasonable aspects in the randomization process and outcome indicators of some studies. ^b^Downgrade one level: The evaluation process of outcome indicators and the follow-up period vary for each study. ^c^Downgrade one level: The evaluation method for outcome indicators is relatively subjective. Low: Limited confidence in effect estimation: true values are likely to be substantially different from estimated values. Very low: We have little confidence in the effect estimate: the true value is likely to be very different from the estimate.

## 4 Discussion

Urticaria is one of the most common allergic skin diseases, with CU representing a more complex and refractory subtype. CU is categorized into chronic spontaneous urticaria and chronic inducible urticaria (4). Mast cell and basophil degranulation are widely recognized as central to the pathogenesis of all CU types. Regarding degranulation, two main mechanisms have been proposed. The first process is not triggered by any identifiable substance and is essentially idiopathic, but involves dysregulation of intracellular signaling pathways within mast cells and basophils. The second involves activation by autoantibodies against FcεRIα or IgE on mast cells and basophils, which may be either immunoglobulin E (IgE)-mediated or non-IgE-mediated (36). Studies show that even the non-lesional skin of CU patients exhibits alterations, such as eosinophil infiltration and increased expression of adhesion molecules and certain cytokines (37, 38). An increasing number of researchers now posit that chronic spontaneous urticaria may have an autoimmune basis, related to an imbalance in the cytokine-chemokine network driven by innate immune response dysregulation (39). The pathogenic mechanism is complex, extending beyond the simple release of vasoactive mediators from mast cells and basophils (40, 41).

The often indeterminable etiology of CU leads to suboptimal treatment responses and high recurrence rates, causing significant distress to patients and severely impacting their quality of life and mental state (42). The current first-line treatment, second-generation H1-antihistamines, often requires long-term use and can cause local or systemic adverse reactions. Overtreatment may worsen the condition (42). More than one-third of patients fail to achieve adequate control with standard or increased doses of second-generation H1-antihistamines, or with combinations of first and second-generation agents (42), highlighting a clear insufficiency for patients with recurrent attacks (5). Alternative treatments such as omalizumab, cyclosporine, and Tripterygium wilfordii preparations are also available; however, some patients remain intolerant or non-responsive, and others experience adverse reactions including gastrointestinal disturbances, renal impairment, and hematopoietic suppression. Furthermore, some newer biologic agents lack high-quality evidence or strong recommendation grades, and their efficacy and safety remain uncertain (4).

Consequently, the exploration of additional treatment options constitutes a significant research direction for CU.

Based on TCM theory, Professor Jin Rui integrated the principles of autohemotherapy with acupuncture to establish AHT. This method combines the effects of bloodletting, acupuncture, and autologous blood injection. Bloodletting and acupuncture are believed to dredge meridians, remove blood stasis, and clear heat, while the injected autologous blood is thought to promote blood circulation, regenerate tissue, and support Qi (43). When administered at specific acupoints, it may further regulate *Zang-fu* organ function. A key advantage of AHT lies in its historical rationale and the clinical efficacy and safety supported by multiple RCTs, particularly for cases where other drug treatments fail or are not tolerated (44). The patient’s autologous blood acts as a personalized, combined antigenic stimulus. Repeated injections may lead to gradual exposure and recognition of antigenic determinants by the immune system. Furthermore, owing to the slow metabolism of autologous blood in the subcutaneous tissue, it provides a mild yet sustained stimulus. This leads to prolonged local immunomodulation at the very site of CU pathology—the subcutaneous layer (44). Various hormones, trace elements, and enzymes present in the blood may not only regulate immune function but also improve microcirculation and skin nutrition, contributing to patient recovery (44).

Acupoint bloodletting therapy may exert its effects by inhibiting the differentiation and proliferation of Th2 cells and modulating humoral immune responses (45). In line with this, a study by Wan-Rong Zhang found that moving cupping combined with autohemotherapy demonstrated superior efficacy and a lower recurrence rate compared to conventional Western medicine for CU, potentially mediated by the downregulation of serum IL-4 and IgE (46).

Electroacupuncture has also shown potential benefits for CU through multiple mechanisms. It can reduce serum IL-4 levels in patients with certain dermatoses, contributing to the alleviation of pruritus (47). Additionally, when applied at acupoints such as Quchi (LI11), Xuehai (SP10), and Zusanli (ST36), electroacupuncture may regulate mast cell degranulation in urticaria models, potentially by suppressing the expression of Lyn and Syk proteins in mast cells, thereby reducing vascular permeability and exerting anti-allergic effects (48).

AHT is frequently combined with other TCM external therapies for managing CU. This combination therapy has demonstrated definite clinical efficacy, a favorable safety profile, and low toxicity.

This study included 20 RCTs (10–29). The meta-analysis results suggest that AHT combined with a subset of other TCM external treatments is superior to Western medicine in terms of overall response rate, recurrence rate, wheal duration, pruritus severity, and serum IgE levels, with statistical significance, and no serious adverse events were reported. The overall response rate results are relatively reliable (except for the acupuncture subgroup). The result for recurrence rate is particularly robust, with low heterogeneity. However, significant heterogeneity persisted for other outcome indicators, despite our best efforts to employ active statistical methods (including subgroup analysis, univariate meta-regression, and sensitivity analysis) to reduce heterogeneity across outcomes. The results for pruritus severity and wheal duration showed significant publication bias, suggesting that the effect sizes for these outcomes may be overestimated. The clinical relevance of changes in serum IgE levels in CU remains uncertain, and this outcome should be interpreted with caution.

Regarding risk of bias, the included studies in our review have the following limitations:① Nine studies (12, 16–18, 22–24, 26, 27) had a high or unclear risk of bias in the randomization process. Although most reported no significant baseline differences between groups, the absence of rigorous randomization and allocation concealment cannot exclude potential confounding from unknown or unmeasured factors. ② Subjective outcomes such as overall response rate and pruritus severity were susceptible to evaluation criteria, assessor judgment, and patient perception. ③ Due to the distinct operational characteristics of TCM external therapies, blinding of patients and investigators was generally difficult to implement. This may introduce a high risk of bias, particularly inflating effect estimates for subjective outcomes such as overall response rate and pruritus severity. Notably, pruritus severity showed high heterogeneity and less robust sensitivity analysis results, whereas the more objective recurrence rate had low heterogeneity. This difference suggests that lack of blinding likely overestimated the effects of subjective outcomes, further reducing the reliability of the evidence.

In addition to the risk of bias, the included studies have the following limitations: ① We only searched for studies published in Chinese or English. All studies were conducted in China, across multiple provinces (e.g., Beijing, Guangdong, Sichuan). Therefore, the applicability of these findings to populations in western China and other countries requires further evaluation. Moreover, studies published in other languages or from other regions may have been missed, which could introduce language and regional publication bias. ② The baseline characteristics of patients were inconsistent, with two studies failing to fully report this information. ③ There were inherent variations in the practical application of TCM external therapies among studies, including differences in acupoint selection, tools, manipulation techniques, treatment duration, and other factors. ④ The follow-up durations, the medications used in the control groups, and the severity and duration of chronic urticaria varied among the included trials. These factors necessitate caution when interpreting the results of this review. ⑤ Outcome assessment criteria were not uniform across studies, and some outcome measures lacked authoritative validation, with varying operational definitions across studies. This inconsistency may increase clinical heterogeneity and hinder accurate interpretation of pooled effect sizes.

Currently, AHT encompasses various techniques such as subcutaneous, intramuscular, whole blood, serum, and acupoint injection. Determining the most effective, simple, and safe method requires large-scale RCTs. Some scholars suggest that circulating autoreactive factors in the blood may have more efficient transfer mechanisms at acupoints compared to non-acupoints, possibly related to the unique distribution of nerve innervation and vascular effects at these sites (47). Furthermore, there is no standardized clinical protocol for AHT combined with various TCM external treatments. Non-standardized operations by untrained personnel in different hospitals could lead to avoidable adverse events. More high-quality RCTs are needed to clarify the efficacy and safety of AHT combined with specific regimens (e.g., loratadine). We urge future investigators to improve methodological quality by applying allocation concealment and blinding where possible, using validated and standardized assessment tools [such as the Urticaria Control Test (UCT) and Urticaria Activity Score over 7 days (UAS7)], emphasizing follow-up studies, and rigorously recording adverse events.

## 5 Conclusion

Existing evidence suggests that AHT combined with most TCM external treatments has good efficacy and a low recurrence rate in treating CU. However, due to the risk of bias and high heterogeneity among the included trials, more rigorous and larger-scale clinical studies are required to further verify the efficacy and safety of this integrative approach.

## Data Availability

The original contributions presented in the study are included in the article/Supplementary material, further inquiries can be directed to the corresponding author.
