# Supplementary material for: Autohemotherapy combined with other external treatments of Traditional Chinese Medicine for chronic urticaria: a systematic review and meta-analysis
Source: Front Med (Lausanne). 2026 Jun 10;13:1818960. doi: 10.3389/fmed.2026.1818960 (PMC13290604; doi:10.3389/fmed.2026.1818960)
Supplement: Supplementary file 2 [file Table_1.docx]

Details of each research trial

| Peng YJ et al.(2023) | ****Intervention Group:**** ****Intervention:**** Acupuncture combined with autohemotherapy. ****1. Acupuncture Procedure:****  ****Acupoints:**** Baihui (GV20), Yintang (EX-HN3), bilateral Fengchi (GB20), Quchi (LI11), Waiguan (TE5), Hegu (LI4), Xuehai (SP10), Zusanli (ST36), and Sanyinjiao (SP6).  ****Needles:**** Disposable sterile acupuncture needles (Hua Tuo brand, 0.30 mm in diameter × 40 mm in length) were used.  ****Needling Technique:****  Baihui (GV20): Subcutaneous insertion of 0.5–0.8 cun.  Yintang (EX-HN3): The local skin was pinched and lifted, followed by a subcutaneous insertion of 0.3–0.5 cun.  Fengchi (GB20): Insertion of 0.8–1.2 cun towards the tip of the nose.  Quchi (LI11), Waiguan (TE5), Hegu (LI4), Xuehai (SP10), Zusanli (ST36), and Sanyinjiao (SP6): Perpendicular insertion of 0.5–1.0 cun.  ****Treatment Schedule:**** Acupuncture was administered 3 times per week (every other day) for a total of 10 weeks.  ****2. Autohemotherapy (AHT) Procedure:****  ****Acupoints:**** Zusanli (ST36) and Xuehai (SP10).  ****Method:**** A 5 mL syringe was used. The median cubital vein was selected as the blood draw site, with a tourniquet applied proximally. After routine disinfection of the venipuncture site and the target acupoints, 4 mL of venous blood was drawn with the needle bevel facing upwards at a 30-degree angle to the skin. The tourniquet was then released, the needle withdrawn, and the puncture site was compressed with a sterile dry cotton swab for 3 minutes. After expelling air from the syringe, the autologous blood was injected perpendicularly into Zusanli (ST36) and Xuehai (SP10) on one side of the body, with 2 mL administered per acupoint. Following injection, the needle was withdrawn, the site was compressed with a dry cotton swab for 1 minute, and covered with a sterile adhesive bandage.  ****Treatment Schedule:**** AHT was performed once per week, with the bilateral Zusanli (ST36) and Xuehai (SP10) acupoints treated on alternating weeks, for a total of 10 weeks.  ****Control Group:**** ****Intervention:**** Oral administration of Loratadine Tablets (Brand name: Clarityne; Specification: 10 mg/tablet; National Medicine Approval Number: H10970410; Manufacturer: Bayer Healthcare Co., Ltd., Shanghai). ****Dosage and Schedule:**** 10 mg was administered orally, once daily, for 10 weeks. |
| --- | --- |
| Zhou GJ et al.(2022) | ****Intervention Group:**** ****Intervention:**** Abdominal Acupuncture combined with Autohemotherapy. ****1. Abdominal Acupuncture Procedure:****  ****Acupoints:**** Zhongwan (CV12), Xiawan (CV10), Qihai (CV6), Guanyuan (CV4), bilateral Huaroumen (ST24), Wailing (ST26), and Daheng (SP15).  ****Needles:**** Disposable sterile filiform needles (0.2 mm in diameter × 40.0 mm in length) were used following routine disinfection.  ****Needling Technique:**** All needles were inserted at the designated acupoints.  ****Needle Retention Time:**** Needles were retained for 30 minutes per session.  ****Treatment Schedule:**** Treatment was administered once every other day. Ten days constituted one treatment course, for a total of 3 courses.  ****2. Autohemotherapy (AHT) Procedure:****  ****Acupoints:**** Bilateral Feishu (BL13) and Quchi (LI11).  ****Method:**** The target acupoints were marked and adequately exposed. Following routine disinfection of the cubital vein area with an iodophor swab, approximately 4 mL of venous blood was drawn. The injection sites were then disinfected with an iodophor swab. The syringe needle was swiftly inserted, and manual stimulation was applied until the patient reported a local needle sensation (deqi) characterized by heaviness, soreness, distension, or numbness. After confirming no blood aspiration upon pulling back the plunger, approximately 1 mL of the autologous blood was slowly injected into each point. The needle was then withdrawn, and the puncture site was compressed with a cotton swab to prevent bleeding.  ****Treatment Schedule:**** The four acupoint pairs (bilateral BL13 and LI11) were used alternately. Treatment was administered once every other day. Ten days constituted one treatment course, for a total of 3 courses.  ****Control Group:**** ****Intervention:**** Oral administration of Loratadine Tablets (Brand name: Clarityne; Tablet, 10 mg/tablet; Manufacturer: Schering-Plough (Shanghai) Pharmaceutical Co., Ltd.; National Medicine Approval Number: H10970410). ****Dosage and Schedule:**** 10 mg was administered orally, once daily, half an hour after breakfast. Ten days constituted one treatment course, for a total of 3 courses. |
| Qin Q(2017) | ****Intervention Group:**** ****Intervention:**** Plum-blossom needle tapping combined with autohemotherapy. ****1. Autohemotherapy (AHT) Procedure:****  ****Acupoints:**** Quchi (LI11), Xuehai (SP10), Sanyinjiao (SP6), and Yinlingquan (SP9).  ****Method:**** The target acupoints were marked and adequately exposed. Following routine disinfection of the cubital vein area with an iodophor solution, approximately 4 mL of venous blood was drawn using a 5 mL disposable syringe. Each injection site was then disinfected with an iodophor swab. The syringe needle was swiftly inserted and manipulated until the patient reported a local needle sensation (deqi) characterized by heaviness, soreness, distension, or numbness. After confirming no blood aspiration upon pulling back the plunger, approximately 1 mL of the autologous blood was slowly injected into each acupoint. The needle was then withdrawn, and the puncture site was compressed with a cotton swab. The same procedure was applied to the remaining acupoints. Patients were instructed to rest for 10 minutes after the procedure.  ****Treatment Schedule:**** AHT was administered once every 3 days. Ten sessions constituted one treatment course.  ****2. Plum-Blossom Needle Tapping Procedure:****  ****Area:**** Bilateral Bladder Meridian pathways on the back, from the level of the first thoracic vertebra (T1) to the fifth lumbar vertebra (L5).  ****Technique:**** Following routine disinfection of the skin area and the plum-blossom needle with 75% alcohol, tapping was performed vertically downward along the designated meridians with a relaxed wrist. Special emphasis was placed on the Pishu (BL20) and Weishu (BL21) acupoints, where tapping was applied more intensively to induce relatively more bleeding. The tapping frequency was maintained at approximately 70-90 times per minute for a duration of 15-20 minutes per session, continuing until the skin became erythematous with minor petechiae.  ****Post-procedure:**** Bleeding points were wiped with a dry cotton ball after tapping.  ****Treatment Schedule:**** Tapping was administered once every other day, for a total of 15 sessions.  ****Control Group:**** ****Intervention:**** Oral administration of Loratadine (Brand name: Clarityne; Manufacturer: Schering-Plough (Shanghai) Pharmaceutical Co., Ltd.; National Medicine Approval Number: H10970410). ****Dosage and Schedule:**** 10 mg was administered orally, once daily at bedtime. Treatment efficacy was assessed upon completion of the 30-day treatment period. |
| Fang LQ et al.(2013) | ****Intervention Group:**** ****Intervention:**** Blood-nourishing and Wind-expelling NeedIing Method combined with autohemotherapy. ****1. Blood-nourishing and Wind-expelling NeedIing Method Procedure:****  ****Acupoints:**** Fengshi (GB31), Fengmen (BL12), Xuehai (SP10), Geshu (BL17), Ganshu (BL18), Pishu (BL20), Sanyinjiao (SP6), Zusanli (ST36), Quchi (LI11), Hegu (LI4), Waiguan (TE5), and Weizhong (BL40). Bilateral acupoints were selected.  ****Needles:**** Disposable sterile filiform needles (0.35 mm in diameter × 50 mm in length).  ****Needling Technique:****  ****Fengshi (GB31):**** Needles were inserted to a depth of 1.5 cun. Following 2 minutes of lifting-thrusting reducing manipulation, needles were retained for 3–5 minutes.  ****Xuehai (SP10):**** Needles were inserted to a depth of 1.2 cun with the needle tip directed towards the sole. Upon achieving deqi (needle sensation), lifting-thrusting and rotating manipulations were applied, with the desired sensation propagating downward. Needles were retained for 30 minutes, with manipulation repeated at 10-minute intervals.  ****Other Acupoints:**** Standard needling techniques specific to each meridian and acupoint were applied.  ****Post-treatment:**** Patients rested for 5 minutes following acupuncture.  ****Treatment Schedule:**** Administered once daily. Seven days constituted one treatment course, with a one-day rest interval between courses.   1. ****Autohemotherapy (AHT) Procedure:****   ****Injection Acupoints:**** Quchi (LI11), Xuehai (SP10), Sanyinjiao (SP6), and Zusanli (ST36).  ****Method:**** Following routine disinfection of the brachial vein area, 8 mL of venous blood was drawn using a 10 mL syringe. The blood was promptly injected into the designated acupoints. After local deqi was elicited and absence of blood aspiration was confirmed upon pulling back the plunger, approximately 2 mL of autologous blood was slowly injected into each acupoint.  ****Injection Protocol:**** To avoid repeat injections on the same side, two acupoints were selected per side during each session, alternating between left and right sides.  ****Treatment Schedule:**** Administered twice per week. Seven days constituted one treatment course, with a one-day rest interval between courses. The total combined treatment duration for both interventions was one month.  ****Control Group:**** ****Intervention:**** Oral administration of Cetirizine Hydrochloride. ****Dosage and Schedule:**** 10 mg was administered orally, once daily. The treatment duration (number of courses) was identical to that of the Intervention Group. |
| Xu J et al.(2005) | ****Intervention Group:**** ****Intervention:**** Acupuncture combined with autohemotherapy. ****1. Acupuncture Procedure:****  ****Acupoint Selection by Syndrome Differentiation:****  ****Wind-Evil Invading Exterior Type:**** Xuehai (SP10), Geshu (BL17), Sanyinjiao (SP6), Quchi (LI11), Hegu (LI4).  ****Gastrointestinal Accumulated Heat Type:**** Zhongwan (CV12), Zusanli (ST36), Hegu (LI4), Shangjuxu (ST37).  ****Yin Deficiency with Effulgent Fire Type:**** Xuehai (SP10), Geshu (BL17), Sanyinjiao (SP6), Zusanli (ST36), Taichong (LR3), Taixi (KI3).  ****Needling Technique:**** For Xuehai (SP10), Geshu (BL17), and Taixi (KI3), a reinforcing manipulation was applied. For all other acupoints, a reducing manipulation was applied.  ****Electroacupuncture:**** After achieving deqi (needle sensation), a G6805 electroacupuncture apparatus was connected to the needles and stimulation was applied for 30 minutes.  ****Treatment Schedule:**** Administered 5 times per week for a total of 4 weeks.  ****2. Autohemotherapy (AHT) Procedure:****  ****Acupoints:**** Quchi (LI11), Zusanli (ST36), Xuehai (SP10), and Feishu (BL13).  ****Method:**** With the patient in a supine position, approximately 10 mL of venous blood was drawn from the median cubital vein after routine disinfection. The autologous blood was injected perpendicularly and swiftly to a depth of 1.0–1.5 cun at the designated acupoints. Following brief lifting-thrusting manipulation to elicit deqi, and after confirming no blood aspiration, approximately 5 mL of blood was slowly injected into each point.  ****Treatment Schedule:**** The above acupoints were used alternately. Treatment was administered once every other day (3 times per week) for a total of 4 weeks.  ****Control Group:**** ****Intervention:**** Oral administration of Chlorphenamine Maleate (4 mg) and Calcium Gluconate (2 g). ****Dosage and Schedule:**** The medications were administered three times daily for a total of 4 weeks. |
| Guo QJ et al.(2024) | ****Intervention Group:**** ****Intervention:**** Du Moxibustion combined with Autohemotherapy. ****1. Du Moxibustion Procedure:****  ****Moxibustion Powder:**** A formulation comprising Huangqi (Radix Astragali), Dangshen (Radix Codonopsis), Jingjie (Herba Schizonepetae), Fangfeng (Radix Saposhnikoviae), Huangqin (Radix Scutellariae), Jianghuang (Rhizoma Curcumae Longae), Difuzi (Fructus Kochiae), Xuchangqing (Radix Cynanchi Paniculati), and Baixianpi (Cortex Dictamni) was prepared in specific proportions, subjected to superfine grinding, and set aside for use.  ****Ginger Paste Preparation:**** Fresh ginger (3 kg) was washed, cut into pieces, and pulverized. Excess juice was partially expressed to achieve a paste of suitable consistency, which was then set aside.  ****Operational Method:****  The participant assumed a prone position on the treatment bed, exposing the back, where the practitioner used a thumbnail to imprint a cross-shaped guideline along the posterior midline and the horizontal line connecting the posterior superior iliac spines. After disinfecting the area along the guideline three times with 75% alcohol swabs, an appropriate amount of ginger juice was applied. Du moxibustion powder was then evenly distributed over the treatment area, followed by placing a strip of mulberry bark paper approximately 6 cm in width and equal in length to the participant's spine over the powder. The ginger paste was evenly spread over the paper, and a central groove was pressed into the paste with a thumb. Olive-shaped moxa cones were placed end-to-end along the groove, with three cones applied consecutively and allowed to burn out completely. Once combustion finished, the moxa ash and ginger paste were removed.  ****Treatment Schedule:**** Administered once every 10 days. Three sessions constituted one treatment course, for a total of 2 courses.  ****2. Autohemotherapy (AHT) Procedure:****  ****Acupoint Groups (bilateral application):****  ****Group 1:**** Quchi (LI11), Zusanli (ST36), Feishu (BL13).  ****Group 2:**** Xuehai (SP10), Geshu (BL17), Pishu (BL20).  ****Method:**** Using a 10 mL sterile syringe, 6 mL of venous blood was drawn from the cubital vein. With the participant positioned to adequately expose the acupoints, each point was disinfected with 75% alcohol. Subsequently, 1 mL of venous blood was injected into each acupoint. The puncture site was compressed firmly following needle withdrawal until hemostasis was achieved.  ****Treatment Schedule:**** The two acupoint groups were used alternately. The treatment cycle consisted of 8 days of intervention followed by a 2-day rest period, constituting one treatment course. A total of 6 courses were administered.  ****Control Group:**** ****Intervention:**** Oral administration of Epinastine Hydrochloride Capsules (Manufacturer: Chongqing Yaoyou Pharmaceutical Co., Ltd.; Batch Number: 13502630; Specification: 10 mg/capsule). ****Dosage and Schedule:**** 10 mg (equivalent to one capsule) was administered orally, once daily. A 30-day period constituted one treatment course, for a total of 2 courses. |
| Kang X(2018) | ****Intervention Group:**** ****Intervention:**** Medicinal Blistering Moxibustion (Tian Jiu) combined with Autohemotherapy.  ****1. Medicinal Blistering Moxibustion (Tian Jiu) Procedure:****  ****Herbal Formula:**** Cangerzi (Fructus Xanthii), Baixianpi (Cortex Dictamni), Jingjie (Herba Schizonepetae), Baishao (Radix Paeoniae Alba), Baijiezi (Semen Sinapis), Gancao (Radix et Rhizoma Glycyrrhizae), Guizhi (Ramulus Cinnamomi), Fangfeng (Radix Saposhnikoviae), and Difuzi (Fructus Kochiae) were ground into fine powder.  ****Paste Preparation:**** 0.05 g of the herbal powder was weighed using a balance and mixed with an appropriate amount of ginger juice to form a medicinal paste.  ****Application:**** The paste was applied to sterile adhesive dressings, which were then affixed to the following acupoints: Waiguan (TE5), Geshu (BL17), Sanyinjiao (SP6), Quchi (LI11), Zusanli (ST36), and Feishu (BL13).  ****Duration:**** Each application lasted for 1 to 2 hours. The dressings were removed once the local skin showed erythema or small blisters.  ****Treatment Schedule:**** Administered once every 10 days, for a total of 4 sessions.  ****2. Autohemotherapy (AHT) Procedure:****  ****Acupoint:**** Zusanli (ST36).  ****Method:**** 5 mL of venous blood was drawn from the patient using a syringe. Following skin disinfection at Zusanli (ST36), the syringe containing the blood was inserted swiftly into the acupoint. Upon the patient experiencing local soreness, numbness, distension, or pain (indicating deqi), the blood was injected.  ****Treatment Schedule:**** Administered twice per week, for a total of 4 sessions.  ****Control Group:**** ****Intervention:**** Oral administration of Mizolastine (Manufacturer: Beijing Honghui Biological Pharmaceutical Co., Ltd.; National Medicine Approval Number: H20020040). ****Dosage and Schedule:**** 10 mg was administered orally, once daily, for a continuous period of 4 weeks. |
| Chen P et al.(2017) | ****Intervention Group:**** ****Intervention:**** Heat-sensitive Moxibustion combined with Autohemotherapy. ****1. Heat-sensitive Moxibustion Procedure:****  ****Acupoints for Exploration:**** Tianshu (ST25), Shenque (CV8), Qihai (CV6), Guanyuan (CV4).  ****Method:**** The patient assumed a comfortable position with the abdomen fully exposed. Two heat-sensitive moxibustion sticks (specification: 2.2 cm × 16.0 cm; Manufacturer: Affiliated Hospital of Jiangxi University of Traditional Chinese Medicine) were ignited. Moxibustion was applied at a distance of 30-50 mm from the skin over the designated acupoints to explore heat-sensitive responses. Throughout the procedure, the practitioner monitored the skin temperature to prevent burns and promptly removed ash from the moxa sticks.  ****Treatment Schedule:**** Each session lasted 45 minutes and was administered once daily. Fifteen days constituted one treatment course, for a total of 2 courses.  ****2. Autohemotherapy Acupoint Injection Procedure:****  ****Acupoints:**** Bilateral Zusanli (ST36).  ****Method:**** The selected acupoints were disinfected. Subsequently, 4 mL of venous blood was drawn from the patient's cubital vein using a 5 mL syringe. A volume of 2 mL was injected into each acupoint. Following needle withdrawal, firm pressure was applied with a sterile cotton swab until bleeding ceased.  ****Treatment Schedule:**** Administered once every 3 days. Five sessions constituted one treatment course, for a total of 2 courses.  ****Control Group:**** ****Intervention:**** Oral administration of Desloratadine Citrate Disodium Tablets (Manufacturer: Guangzhou Hairui Pharmaceutical Co., Ltd.; Specification: 8.8 mg/tablet). ****Dosage and Schedule:**** 8.8 mg was administered orally once daily at bedtime. Fifteen days constituted one treatment course, for a total of 2 courses. |
| Su KX(2020) | ****Intervention Group:**** ****Intervention:**** Catgut embedding therapy combined with autohemotherapy. ****1. Autohemotherapy (AHT) Procedure:****  ****Acupoints:**** Bilateral Zusanli (ST36).  ****Method:**** The treatment area was routinely disinfected. Approximately 2 mL of venous blood was drawn from the median cubital vein using a disposable syringe. The syringe needle was swiftly inserted subcutaneously. Mild lifting-thrusting (without rotation) was applied until the patient reported a local deqi sensation (soreness, numbness, distension, or heaviness). After confirming no blood aspiration upon pulling back the plunger, 1 mL of the blood was rapidly injected to prevent coagulation. Upon completion, the puncture site was compressed with a sterile cotton swab. The same procedure was then performed on the contralateral Zusanli (ST36).  ****Treatment Schedule:**** Administered once per week. Four sessions constituted one treatment course. Efficacy was assessed after completing 2 courses.  ****2. Catgut embedding therapy Procedure:****  ****Acupoints:**** Bilateral Xuehai (SP10), Geshu (BL17), Sanyinjiao (SP6), Quchi (LI11), and Tianshu (ST25).  ****Method:**** The skin over the acupoints was routinely disinfected with iodophor. A pre-cut segment of catgut was placed into a No. 6 injection needle bore using sterile forceps. The position of a custom-made stylet was adjusted to ensure the catgut was completely contained within the needle and not exposed externally. Holding the needle in the right hand, it was inserted to the required depth. Upon eliciting local deqi, the stylet was advanced using a filiform needle held in the left hand, while simultaneously withdrawing the needle barrel slowly with the right hand. When a distinct loss of resistance was felt, indicating the catgut had been deposited into the subcutaneous or muscular layer, the needle barrel was swiftly and completely withdrawn. The puncture site was immediately compressed with a cotton swab.  ****Treatment Schedule:**** Administered once every 2 weeks. Two sessions constituted one treatment course. Efficacy was assessed after completing 2 courses.  ****Control Group:**** ****Intervention:**** Oral administration of Loratadine Dispersible Tablets (Specification: 10 mg/tablet; Manufacturer: Beijing Double-Crane Pharmaceutical Co., Ltd.; National Medicine Approval Number: H20030208). ****Dosage and Schedule:**** One tablet (10 mg) was administered orally, once daily. A 28-day period constituted one treatment course. Following 2 weeks of continuous administration, there was a 2-week drug-free interval before commencing the second course. A total of 2 courses were administered. |
| Xie XP et al.(2017) | ****Treatment Group:**** ****Intervention:**** Catgut embedding therapy combined with autohemotherapy. ****1. Catgut embedding therapy Procedure:****  ****Primary Acupoints:**** Bilateral Hegu (LI4), Taichong (LR3), Sanyinjiao (SP6), Quchi (LI11), Xuehai (SP10), Weizhong (BL40), and Zusanli (ST36).  ****Supplementary Points Based on Syndrome Differentiation:****  ****Wind-Heat Type:**** Fengchi (GB20), Dazhui (GV14).  ****Gastrointestinal Accumulated Heat Type:**** Zusanli (ST36), Tianshu (ST25).  ****Deficiency of both Qi and Blood Type:**** Qihai (CV6), Zusanli (ST36).  ****Method:**** The local acupoint sites were routinely disinfected. A segment of 4-0 catgut was loaded into a No. 7 needle barrel, and the stylet was connected. The needle was swiftly inserted into the acupoint. The insertion depth and the length of the catgut segment were adjusted according to the local anatomy, with the catgut intended to traverse the fascial layer. After withdrawing the needle, the puncture site was compressed and disinfected with a sterile cotton ball.  ****2. Autohemotherapy (AHT) Procedure:****  ****Acupoints:**** Dazhui (GV14) and bilateral Geshu (BL17), Feishu (BL13), Quchi (LI11), and Zusanli (ST36).  ****Method:**** 5–10 mL of venous blood was drawn from the cubital vein. The blood was then promptly injected into the designated acupoints using the syringe, with 2–3 mL administered per point.  ****Combined Treatment Schedule:**** Acupoint catgut embedding was performed once every 15 days. AHT was administered once daily for 10 consecutive days.  ****Control Group:**** ****Intervention:**** Conventional Western medication. ****Regimen:****  Oral Cetirizine Hydrochloride: 10 mg once daily.  Oral Ebastine: 10 mg once daily.  ****Treatment Duration:**** Continuous administration for one month. |
| Zhang XX(2015) | ****Treatment Group:**** ****Intervention:**** Catgut embedding therapy combined with autohemotherapy. ****1. Autohemotherapy (AHT) Procedure:****  ****Acupoints:**** Quchi (LI11) and Xuehai (SP10).  ****Method:**** 10 mL of autologous venous blood was drawn using a 10 mL syringe. Approximately 2–3 mL of blood was injected into each acupoint.  ****Treatment Schedule:**** Administered twice per week. Ten sessions constituted one treatment course.  ****2. Catgut embedding therapy Procedure:****  ****Acupoints:**** Feishu (BL13), Fengmen (BL12), Pishu (BL20), Ganshu (BL18), Shenshu (BL23), Quchi (LI11), Xuehai (SP10), Zusanli (ST36), and Yinlingquan (SP9).  ****Method:**** The puncture needle embedding method was employed. Four to six acupoints were selected per session and used alternately. The selected sites were routinely disinfected. A 1 cm segment of No. 4 catgut was grasped with forceps and placed into the barrel of a No. 7 hypodermic needle. The needle was swiftly inserted into the acupoint. For Back-Shu points (BL13, BL12, BL20, BL18, BL23), the needle was inserted obliquely towards the spine. Gentle lifting-thrusting manipulation was applied until the patient reported sensations of soreness, numbness, or distension (deqi). A 75 mm long acupuncture needle (0.35 mm diameter) was then used to push the catgut segment into the subcutaneous tissue or muscular layer beneath the acupoint, ensuring no part of the catgut protruded from the skin. The puncture site was disinfected and covered with an adhesive bandage for protection.  ****Treatment Schedule:**** Administered once every 10 days. Five sessions constituted one treatment course.  ****Control Group:**** ****Intervention:**** Oral administration of Desloratadine Citrate Disodium Tablets (Brand name: Beixue; Manufacturer: Yangtze River Pharmaceutical Group Guangzhou Hairui Pharmaceutical Co., Ltd.). ****Dosage and Schedule:**** 8.8 mg was administered orally, once daily, for a total of 30 days. |
| Chen BF et al.(2022) | ****Comprehensive Group:**** ****Intervention:**** Acupoint-application combined with autohemotherapy. ****1. Autohemotherapy (AHT) Procedure:****  ****Acupoints:**** Dingchuan (EX-B1), Quchi (LI11), and Zusanli (ST36).  ****Method:**** The patient assumed an upright sitting position. Following routine disinfection of the skin over the cubital area, 3 mL of venous blood was drawn from the cubital vein using a disposable 5 mL syringe. The blood was immediately injected into the disinfected Dingchuan (EX-B1), Quchi (LI11), and Zusanli (ST36) points on one side, with 1 mL administered per point. Upon completion, the puncture sites were compressed with cotton swabs to achieve hemostasis.  ****Treatment Schedule:**** Bilateral acupoints were used alternately. Treatment was administered once every 3 days, for a total of 14 sessions over a 6-week treatment period.  ****2. Acupoint-application Procedure:****  ****Acupoint:**** Shenque (CV8).  ****Herbal Formula (Xiao Feng San):**** The formula consisted of Danggui (Radix Angelicae Sinensis) 9 g, Shengdi (Radix Rehmanniae) 9 g, Humaren (Semen Sesami Nigrum) 9 g, Fangfeng (Radix Saposhnikoviae) 9 g, Jingjie (Herba Schizonepetae) 9 g, Chantui (Periostracum Cicadae) 9 g, Niubangzi (Fructus Arctii) 9 g, Cangzhu (Rhizoma Atractylodis) 9 g, Kushen (Radix Sophorae Flavescentis) 9 g, Mutong (Caulis Akebiae) 6 g, Shigao (Gypsum Fibrosum) 9 g, Zhimu (Rhizoma Anemarrhenae) 9 g, and Gancao (Radix et Rhizoma Glycyrrhizae) 6 g.  ****Preparation and Application:**** The above herbs were ground into an extremely fine powder. For each application, a small amount of the powder was mixed with honey to form a paste, which was then applied to the routinely disinfected Shenque (CV8) acupoint.  ****Treatment Schedule:**** Each application lasted for 6 hours. Treatment was administered once every 3 days, for a total of 14 sessions over a 6-week treatment period.  ****Control Group:**** ****Intervention:**** Oral administration of Ebastine Tablets (Brand name: Kestine; Manufacturer: Almirall S.A., Spain; Registration Certificate Number: H20140855; Specification: 10 mg/tablet). ****Dosage and Schedule:**** One tablet (10 mg) was administered orally, once daily, for a total of 6 weeks. |
| Chen BF(2019) | ****Comprehensive Group:**** ****Intervention:**** Acupoint-application combined with autohemotherapy. ****1. Autohemotherapy (AHT) Procedure:****  ****Acupoints:**** Dingchuan (EX-B1), Quchi (LI11), and Zusanli (ST36) on the same side.  ****Method:**** The patient assumed a sitting position. The acupoints on one side were located, marked, and adequately exposed. Following standard venipuncture protocol, 3 mL of venous blood was drawn from the median cubital vein using a disposable 5 mL sterile syringe (Brand: Yusheng; Specification: 5 mL/unit; Manufacturer: Zhejiang Yusheng Co., Ltd.; Production License No.: 20100192). The exposed acupoints on the same side were then routinely disinfected. The syringe needle was swiftly inserted approximately 0.5 cun into each acupoint. After confirming no blood aspiration, 1 mL of blood was slowly injected into each point. Upon completion, the puncture sites were compressed with medical cotton swabs (Specification: 20 pieces/pack; Manufacturer: Yixin Medical Devices Co., Ltd.; Production License No.: 20160050) to prevent bleeding.  ****Treatment Schedule:**** Bilateral sides were treated alternately. The procedure was performed once every 3 days, for a total of 14 sessions over 6 weeks.  ****2. Acupoint-application Procedure:****  ****Acupoint:**** Shenque (CV8).  ****Herbal Formula Composition:**** Danggui (Radix Angelicae Sinensis) 10 g, Baishao (Radix Paeoniae Alba) 15 g, Shengdihuang (Radix Rehmanniae) 15 g, Chuanxiong (Rhizoma Chuanxiong) 10 g, Huangqi (Radix Astragali) 20 g, Zhiheshouwu (Radix Polygoni Multiflori Praeparata) 10 g, Jingjie (Herba Schizonepetae) 10 g, Fangfeng (Radix Saposhnikoviae) 10 g, Cijili (Fructus Tribuli) 10 g, Gancao (Radix et Rhizoma Glycyrrhizae) 6 g.  ****Preparation and Application:**** All herbs were ground into an extremely fine powder. For each application, an appropriate amount of the powder was mixed with sterile water for injection to form a paste. The paste was evenly spread into the central groove of a medical acupoint application patch (with a leak-proof ring, inner diameter: 2.5 cm, height: 0.3 cm, patch size: 7 cm × 8 cm). After routine disinfection of the Shenque (CV8) acupoint, the patch was firmly applied to the umbilicus. Participants were instructed to secure the patch to prevent detachment and to remove it themselves after 6 hours of continuous application.  ****Treatment Schedule:**** Applied once every 3 days, for a total of 14 sessions over 6 weeks.  ****Control Group:**** ****Intervention:**** Oral administration of Ebastine Tablets (Brand name: Kestine; Specification: 10 mg × 10 tablets/box; Manufacturer: Almirall S.A., Spain; Production License No.: H20090366). ****Dosage and Schedule:**** 10 mg was administered orally, once daily, for a total of 6 weeks. |
| Liang LQ et al.(2020) | ****Intervention Group:**** ****Intervention:**** Auricular apex bloodletting combined with autohemotherapy. ****1. Auricular Apex Bloodletting Procedure:****  ****Material Preparation:**** Sodium bicarbonate was placed in an aluminum spoon and heated over low heat until completely dry, producing anhydrous sodium bicarbonate, which was stored in a sterile vial for later use.  ****Operational Method:**** The bilateral auricular apex acupoints were routinely disinfected with iodophor followed by alcohol. Local infiltration anesthesia was administered using 0.5–1.0 mL of 2% lidocaine. A sterile surgical scalpel was used to make a sagittal incision approximately 4–5 mm in length and 0.5–1.0 mm in depth at the auricular apex, until bleeding occurred. Sterile cotton balls were used to continuously wipe away the blood until it stopped. Finally, a small amount of anhydrous sodium bicarbonate was placed into the incision, covered with a sterile dressing, and secured with adhesive tape.  ****2. Autohemotherapy (AHT) Procedure:****  ****Acupoints:**** Quchi (LI11) and Xuehai (SP10).  ****Method:**** Using a 5 mL syringe, 4 mL of the patient's own venous blood was drawn. Following routine skin disinfection, the blood was injected into the designated acupoints, with 2 mL administered per point.  ****Treatment Schedule:**** Administered once daily, 4 consecutive days per week, for a total treatment duration of 4 weeks. If symptoms persisted after one course, a second course could be initiated after a 2-week interval.  ****Control Group:**** ****Intervention:**** Western medication therapy.  ****Medication 1:**** Loratadine Tablets (National Medicine Approval Number: H20070030; Manufacturer: Xi'an Janssen Pharmaceutical Ltd.; Specification: 10 mg/tablet). ****Dosage and Schedule:**** 10 mg (one tablet) was administered orally, once daily, half an hour after breakfast.  ****Medication 2:**** Ketotifen Tablets (National Medicine Approval Number: H33020917; Manufacturer: Hangzhou Supor Nanyang Pharmaceutical Co., Ltd. (formerly Zhejiang Nanyang Pharmaceutical Co., Ltd.); Specification: 1 mg/tablet). ****Dosage and Schedule:**** 10 mg was administered orally, once daily, half an hour before bedtime. |
| Zhang P et al.(2021) | ****Intervention Group:**** ****Intervention:**** Moving cupping therapy combined with autohemotherapy.  ****1. Autohemotherapy (AHT) Procedure:****  ****Blood Collection:**** Venous blood was drawn from the cubital transverse crease.  ****Injection Acupoints:**** Quchi (LI11) and Xuehai (SP10), alternated weekly.  ****Method:**** 2 mL of blood was injected per acupoint to an approximate depth of 1.0–1.5 cun. Following injection, the puncture site was compressed for 3–5 minutes until hemostasis was achieved.  ****Post-procedure:**** Patients rested for 20 minutes.  ****2. Moving Cupping Procedure:****  ****Treatment Area:**** The Bladder Meridian of Foot-Taiyang and the Governor Vessel (Du Meridian) on the back.  ****Position:**** Prone.  ****Method:**** An appropriate amount of massage oil was applied along the meridian pathways. Moving cupping was performed from Dazhui (GV14) downwards, repeated 3–4 times until the skin became erythematous with petechiae.  ****Treatment Schedule:**** Sessions were conducted at 5–7 day intervals. One month constituted one treatment course.  ****Control Group:**** ****Intervention:**** Cetirizine tablets combined with Dexamethasone Acetate Cream.  ****Oral Medication:**** Cetirizine Tablets (Brand name: Xili; National Medicine Approval Number: H19980059; Manufacturer: Yangtze River Pharmaceutical Group Co., Ltd.; Specification: 10 mg/tablet). ****Dosage and Schedule:**** 10 mg administered orally, once daily.  ****Topical Medication:**** Dexamethasone Acetate Cream (Brand name: Piyanping; National Medicine Approval Number: H44024170; Manufacturer: China Resources Sanjiu Medical & Pharmaceutical Co., Ltd.). ****Application:**** The cream was applied evenly to pruritic areas, twice daily.  ****Treatment Course:**** One month. |
| Zhao HN et al.(2021) | ****Intervention Group:**** ****Intervention:**** Cupping therapy combined with autohemotherapy. ****1. Autohemotherapy (AHT) Procedure:****  ****Acupoints:**** Bilateral Zusanli (ST36) and Sanyinjiao (SP6).  ****Method:**** 4 mL of cubital venous blood was drawn using a disposable syringe. Subsequently, 1 mL of the autologous venous blood was injected into each of the four acupoints. Immediately after injection, the puncture site was compressed with a cotton swab or covered with an adhesive bandage.  ****Treatment Schedule:**** Administered once every 3 days. Two sessions constituted one treatment course, for a total of 4 courses.  ****2. Cupping therapy Procedure:****  ****Acupoint:**** Shenque (CV8).  ****Method:**** Cupping was applied to Shenque (CV8) using the flash-fire method. Each application was retained for 2 minutes before removal. This process (application-retention-removal) was repeated twice more, resulting in a total of 3 cycles and a cumulative cupping duration of 6 minutes per session.  ****Treatment Schedule:**** Administered once every other day. Three sessions constituted one treatment course, for a total of 4 courses.  ****Control Group:**** ****Intervention:**** Oral administration of Levocetirizine (Manufacturer: Chongqing Huapont Pharmaceutical Co., Ltd.; National Medicine Approval Number: H20040249). ****Dosage and Schedule:**** 5 mg was administered orally, once daily. Seven days constituted one treatment course, for a total of 4 courses. |
| Zhang WR et al.(2014) | ****Intervention Group:**** ****Intervention:**** Moving cupping therapy combined with autohemotherapy. ****1. Autohemotherapy (AHT) Procedure:****  ****Acupoints:**** Bilateral Quchi (LI11) and Zusanli (ST36).  ****Method:**** Under aseptic conditions, 4 mL of the patient's own venous blood was drawn. The acupoints were routinely disinfected. The syringe needle was swiftly inserted into each acupoint. Once the patient reported a local sensation of soreness, distension, or numbness (deqi), and after confirming no blood aspiration, 1 mL of blood was rapidly injected into each point. The needle was then withdrawn, and the puncture site was compressed with a disinfected cotton swab.  ****Post-procedure:**** Patients rested for 10 minutes.  ****2. Moving Cupping Procedure:****  ****Position:**** Prone.  ****Area:**** The Governor Vessel (Du Meridian) and the Bladder Meridian of Foot-Taiyang pathways.  ****Method:**** An appropriate amount of petroleum jelly was evenly applied to the meridian pathways. A cup was quickly applied to Dazhui (GV14) using the flash-fire method. The cup was then moved by hand along the designated meridians, from Dazhui (GV14) superiorly to Shenshu (BL23) inferiorly, with 3–4 upward and downward passes, until the skin became reddish-purple, congested, or even exhibited petechiae.  ****Combined Treatment Schedule:**** The two methods were used in combination. Treatment was administered once every 3 days, for a total of 10 sessions over 30 days.  ****Control Group:**** ****Intervention:****  ****Oral Medication:**** Cetirizine Tablets (Brand name: Xili; National Medicine Approval Number: H19980059; Manufacturer: Yangtze River Pharmaceutical Group Co., Ltd.; Specification: 10 mg/tablet). ****Dosage:**** 10 mg orally, once daily.  ****Topical Medication:**** Compound Dexamethasone Acetate Cream (Brand name: Piyanping; National Medicine Approval Number: H44024170; Manufacturer: China Resources Sanjiu Medical & Pharmaceutical Co., Ltd.). ****Application:**** Applied topically, 1–2 times daily.  ****Treatment Duration:**** 30 days. |
| Ding Y(2014) | ****Intervention Group:**** ****Intervention:**** Cupping therapy combined with autohemotherapy.  ****1. Cupping Procedure:****  ****Position:**** Supine, with the umbilical area exposed.  ****Acupoint:**** Shenque (CV8).  ****Method:**** The flash-fire method was used to apply and remove the cup rapidly over Shenque (CV8) 4–5 times until the local skin became flushed. Subsequently, the cup was firmly placed over Shenque (CV8) using the flash-fire method and retained for 5–10 minutes (retention time was adjusted based on patient tolerance; a small amount of air could be released if discomfort occurred).  ****Treatment Schedule:**** Administered once every 3 days. Ten sessions constituted one treatment course (equivalent to 4 weeks).  ****2. Autohemotherapy (AHT) Procedure:****  ****Acupoint Groups (bilateral):****  ****Group A:**** Geshu (BL17) and Feishu (BL13).  ****Group B:**** Quchi (LI11) and Xuehai (SP10).  ****Selection Protocol:**** Group A was used for the first session. Groups A and B were used alternately thereafter.  ****Method:****  The practitioner performed routine disinfection over the cubital vein area using a cotton swab and drew 4 mL of venous blood.  The selected acupoints were located and routinely disinfected.  The syringe containing venous blood was swiftly inserted into the acupoint, initially injecting approximately 0.5–0.7 mL.  After eliciting deqi (needle sensation), the plunger was pulled back slightly. If blood aspiration was difficult, the autologous blood was injected rapidly. If aspiration was easy or resistance was met during injection, the needle direction was adjusted to facilitate easier injection, followed by blood administration.  After injection, the needle was swiftly withdrawn. The puncture site was compressed with a disinfected cotton swab and then covered with an adhesive bandage to prevent leakage.  ****Needling Notes:****  ****Back Acupoints (BL17, BL13):**** The needle was inserted obliquely and slightly medially towards the spine to a depth of approximately 0.5–0.8 cun. Deep insertion was avoided to prevent pneumothorax.  ****Limb Acupoints (LI11, SP10):**** The needle was inserted perpendicularly to a depth of approximately 1.0 cun. After achieving deqi, 1 mL of blood was injected per point.  ****Treatment Schedule:**** Administered once every 3 days. Ten sessions constituted one treatment course (equivalent to 4 weeks).  ****Control Group:**** ****Intervention:**** Oral administration of Loratadine (Brand name: Clarityne; Specification: 10 mg/tablet; Manufacturer: Schering-Plough (Shanghai) Pharmaceutical Co., Ltd.; National Medicine Approval Number: H10970410). ****Dosage and Schedule:**** One tablet (10 mg) was administered orally, once daily, for 4 consecutive weeks. |
| Zhang YH et al.(2017) | ****Treatment Group:**** ****Intervention:**** Acupoint Bloodletting Cupping combined with autohemotherapy.  ****1. Acupoint Bloodletting Cupping Procedure:****  ****Acupoints (bilateral):**** Feishu (BL13), Fengmen (BL12), and Xuehai (SP10).  ****Method:**** The acupoints were routinely disinfected. Each point was swiftly pricked approximately 10 times using a three-edged needle. A No. 2 glass cup was then applied over each pricked point for 10 minutes, resulting in a bloodletting volume of 5–10 mL per cup. Bleeding was generally allowed to stop spontaneously. If a point showed no or minimal bleeding after cup removal, it was re-pricked more vigorously with the three-edged needle before re-applying the cup for another 10 minutes. After final cup removal, coagulated blood was wiped away and the skin was disinfected to prevent infection.  ****2. Autohemotherapy (AHT) Procedure:****  ****Acupoints:**** Bilateral Zusanli (ST36).  ****Method:**** 4 mL of autologous venous blood was drawn from the cubital vein using a 5 mL disposable syringe. After needle insertion at Zusanli (ST36), lifting-thrusting manipulation was performed until the patient felt soreness and distension. Upon confirming no blood aspiration, 2 mL of blood was rapidly injected into each acupoint.  ****Treatment Schedule:**** Administered once every 3 days. A total of 20 sessions over 60 days constituted one treatment course.  ****Control Group:**** ****Intervention:**** Oral administration of Loratadine Dispersible Tablets (Manufacturer: Beijing Double-Crane Pharmaceutical Co., Ltd.; Specification: 10 mg × 6 tablets; Batch Number: 20131001). ****Dosage and Schedule:**** 10 mg was administered orally, once daily. Continuous administration for 60 days constituted one treatment course. |
| Jia YN et al.(2020) | ****Catgut embedding therapy and Cupping Group:**** ****Intervention:**** Catgut embedding therapy and Cupping combined with autohemotherapy. ****1. Catgut embedding therapy and Cupping Procedure:****  ****Acupoints (bilateral):**** Feishu (BL13), Xinshu (BL15), Geshu (BL17), Ganshu (BL18), and Sanjiaoshu (BL22).  ****Position:**** Prone.  ****Method:****  The acupoints were located and routinely disinfected with iodophor.  Collagen threads, pre-soaked in 75% ethanol, were cut into 1–2 cm segments, placed in a sterile container, rinsed with normal saline, and then loaded into disposable No. 9 sterile thread-embedding needles using sterile forceps.  The needle was slowly inserted into the selected acupoint. Upon the patient experiencing deqi sensations (soreness, numbness, distension, or pain), the stylet was gently and slowly advanced to deposit the collagen thread into the muscular layer beneath the acupoint. The needle was then withdrawn, and the site was disinfected with iodophor.  After completing thread-embedding at all acupoints, a fire cup was applied over each embedded site for 5 minutes.  Following cup removal, the area was disinfected again with iodophor and covered with a bottle orifice dressing for 2 hours.  ****2. Autohemotherapy (AHT) Procedure:****  ****Acupoint:**** Bilateral Zusanli (ST36).  ****Method:**** The patient assumed a supine position. Following standard venipuncture protocol, 4 mL of venous blood was drawn using a 5 mL disposable syringe. The acupoints were routinely disinfected. The syringe needle was swiftly inserted into bilateral Zusanli (ST36). Upon eliciting local deqi and confirming no blood aspiration, 2 mL of blood was injected into each side. The needle was withdrawn, and the puncture site was covered with a bottle orifice dressing.  ****Treatment Schedule:**** Administered once per week. Three weeks constituted one treatment course, for a total of 3 courses.  ****Plum-blossom Needle Cupping Therapy Group:**** ****Intervention:**** Plum-blossom Needle Cupping Therapy combined with autohemotherapy. ****1. Plum-blossom Needle Cupping Therapy Procedure:****  ****Acupoints (bilateral):**** Feishu (BL13), Xinshu (BL15), Geshu (BL17), Ganshu (BL18), and Sanjiaoshu (BL22).  ****Position:**** Prone, with the back and Bladder Meridian exposed.  ****Method:**** The acupoint areas were disinfected with iodophor swabs. A plum-blossom needle (Manufacturer: Suzhou Medical Supplies Co., Ltd.) was used to tap the acupoints until slight bleeding occurred, within the patient's tolerance. Fire cups were then immediately applied over the tapped areas for 5 minutes. After cup removal, the local area was cleaned and routinely disinfected.  ****2. Autohemotherapy (AHT) Procedure:****  The AHT procedure (acupoints, method, treatment schedule, and precautions) was identical to that described for the Thread-embedding & Cupping Group.  ****Control Group:**** ****Intervention:**** Oral administration of Ebastine Tablets (Manufacturer: Jiangsu Lianhuan Pharmaceutical Co., Ltd.; National Medicine Approval Number: H20040119; Specification: 10 mg/tablet). ****Dosage and Schedule:**** One tablet (10 mg) was administered orally, once daily. Three weeks constituted one treatment course, for a total of 3 courses. |

a,1 cun (Chinese inch) ≈ 3.33 cm ≈ 1.31 inches.
